# TailCoR: A new and simple metric for tail correlations that disentangles the linear and nonlinear dependencies that cause extreme co-movements

**DOI:** 10.1371/journal.pone.0278599

**Published:** 2023-01-03

**Authors:** Sladana Babić, Christophe Ley, Lorenzo Ricci, David Veredas

**Affiliations:** 1 LeasePlan, Amsterdam, The Netherlands; 2 Department of Mathematics, University of Luxembourg, Esch-sur-Alzette, Luxembourg; 3 European Stability Mechanism, Luxembourg, Luxembourg; 4 Centre for Sustainable Finance and Department of Economics, Vlerick Business School and Ghent University, Brussels, Belgium; SGH Warsaw School of Economics: Szkola Glowna Handlowa w Warszawie, POLAND

## Abstract

Economic and financial crises are characterised by unusually large events. These tail events co-move because of linear and/or nonlinear dependencies. We introduce TailCoR, a metric that combines (and disentangles) these linear and non-linear dependencies. TailCoR between two variables is based on the tail inter quantile range of a simple projection. It is dimension-free, and, unlike competing metrics, it performs well in small samples and no optimisations are needed. Indeed, TailCoR requires a few lines of coding and it is very fast. A Monte Carlo analysis confirms the goodness of the metric, which is illustrated on a sample of 21 daily financial market indexes across the globe and for 20 years. The estimated TailCoRs are in line with the financial and economic events, such as the 2008 great financial crisis and the 2020 pandemic.

## 1 Introduction

Two major global crises have stricken the economy and financial markets since 2000: The 2007–2008 Great Financial Crisis and the 2020 Great Lockdown due to COVID-19. These crises highlight the importance of tail—or rare—events that can have different natures: corporate and Government defaults, stock market crashes, public health emergencies, or political decisions, to name a few.

When they occur, their shock waves spread over the economic and financial systems through large co-movements. These unfrequent and joint events locate on the tails of the probability distributions. From a statistical point of view, joint tail events are caused by linear and/or nonlinear correlations. Indeed, correlations on the tails may happen because variables are linearly correlated (i.e., the Pearson correlations are different from zero) and/or nonlinearly correlated, in the sense that variables are dependent at the tails of their distributions only. Other forms of nonlinearity may occur but we do not take them into account. They can however be considered within the theory of TailCoR. An illustrative example is a multivariate Student-t distribution, where the dependence between the random variables is through the linear correlations and the tail index.

Several statistical measures have been proposed to assess the degree of association on the tails. The tail dependence coefficients (also called extremal dependence structures and co-exceedance probabilities) stemming from extreme value theory (EVT henceforth) are the most used. These coefficients rely on two building blocks. The first is the assumption that tails of the distribution (either the joint and/or the marginal distributions) asymptotically decay according to a power law (see [1, 2] for references on the multivariate generalized Pareto distribution).

The second building block is the extreme value copula (also called stable tail dependence function or STDF), see e.g. [3–6]. Broadly speaking, estimation of the STDF is divided in two approaches: parametric and semi-parametric. The former is based on parametric copula functions, the logistic and *t* copulas are commonly used (see, for example, [7] for its use in investment diversification across major stock market indexes). The latter is based on higher order statistics (see [4] for a theoretical treatment, and [8] for an application to asset market linkages in crisis periods).

[9] exploit, in the bivariate case, the explicit and simple relation between the tail dependence coefficient of the Gumbel copula and the linear correlation coefficient for extreme events, also known as exceedance correlation. Their application is in international equity markets. In a similar vein, [10] introduce the quantile correlation, i.e. the sample correlation between observations that are contained in a ball around a given joint quantile. The measures of [9, 10] share that when applied to thresholds and quantiles that are far on the tails, the number of observations is limited, and large sample sizes are needed to obtain precise estimators. Moreover, these measures are not able to disentangle between the linear and nonlinear contributions.

As pointed out in [11], estimation of the STDF has the weakness that it presupposes tail dependence, besides that the parametric copula rests on parametric assumptions. An alternative proposed by [12] consists of testing for tail dependence through the tail index of an auxiliary variable (the cross-sectional minimum of the random vector). [13] uses it for studying the banking system, [14] for estimating the tail dependence among risky asset returns, and [11] for studying US sectoral stock indexes around 9/11, respectively.

The above mentioned work on EVT, though relevant and important, has shortcomings. The main one is that the theory builds upon asymptotic results on the tails. Since in practice we estimate using a finite cut-off point of the tail, the asymptotic results are an approximation. Moreover, precise estimators require large sample sizes.

While EVT-based literature is semi-parametric (in the sense that only the asymptotic tail is parametrized), another branch of the literature parametrises the whole distribution while assuming heavy tails—see [15] for a survey. The scale mixtures of multinormal distributions are particularly relevant since the elliptical and the normal mean-variance mixture distributions are used in this article. These distributions have the feature that the dependence between two random variables is given by the dispersion matrix and a single tail index (and the vector of skewness in the case of the normal mean-variance mixture). [16] proposes a new family of distributions, coined multiple scaled distributions, with the main feature that there are as many tail indexes as the dimension of the random vector. Another relevant fully parametric class of distributions are copulas, where the meta-elliptical copula of [17] plays a dominant role. Beyond ellipticity, [18] propose a multivariate conditional variance model that allows to model conditional correlation and dependence separately and simultaneously with nonelliptically distributed dependent errors. While fully parametrising the whole distribution oftentimes allows to disentangle between the linear and nonlinear correlations, the analytical form of these correlations heavily depends on the distributional choice. Moreover, in large dimensional problems only the Gaussian (which does not have tail dependence) and Student-*t* copulas are realistic and feasible, while in vast dimensional problems only the Gaussian is feasible.

TailCoR overcomes the shortcomings of EVT and fully parametric measures. TailCoR is simple, it does not suffer form the curse of dimensionality, it performs well in small samples, and it disentangles the above mentioned linear and nonlinear correlations under the very mild assumption that the probability contours are elliptical. Moreover, it can be computed under tails that are fatter, equal, or thinner than Gaussian.

In a nutshell, TailCoR between two random variables is based on the projection of the variables onto a line, and then a tail Inter Quantile Range (IQR) is computed. Since the projection is computed as a sum of a cosine and a sine, and the IQR is straightforward, TailCoR does not require optimizations and the coding merely consists of a dozen of lines.

We illustrate TailCoR on daily returns of 21 financial market indexes across the globe. Data runs from January 2000 to July 2020, and hence it incorporates the two crises mentioned earlier. For comparison purposes, we compute the upper and lower exceedance correlations of [9], and the parametric and non-parametric tail dependence coefficients (the former with a *t* copula, and the latter as in [11]). Full sample results show geographical clusters, especially in North America and Europe. The clusters are driven by the linear correlations, which relate to the investment freedom of the countries where the markets operate. We do not find clustering of the nonlinear correlations, meaning that during periods of extreme events, contagion spreads, causing extreme co-movements.

To study the dynamic behaviour we use rolling windows of 3 years. The estimated TailCoRs are in line with the financial and economic events that happened during the sample. TailCoR increases in crises periods, when global dependence increases, as it happened in 2007–2008 (and the aftermath) as well as recently because of COVID-19. The evolution of the other metrics is heterogeneous. While the exceedance correlations could not be computed because of lack of extreme observations in the windows, the tail dependence coefficients move along with TailCoR though they show a greater deal of variability.

The remaining sections are laid out as follows. Section 2 introduces the notation, assumptions, definition, and representations of TailCoR. It also shows the asymptotic properties of the estimators. Section 3 covers a brief Monte Carlo study. The illustration to the market indexes is presented in Section 4, while Section 5 concludes, and proofs are relegated to the S1 Appendix. A separate S2 Appendix includes lengthy tables that is available for consultation.

## 2 TailCoR

### 2.1 Definition

Let **X**_*t*_, *t* = 1, …, *T*, be a random vector of size *N* at time *t* satisfying the following assumption

**G1** (a) The random process {**X**_*t*_} is a strongly stationary sequence of random vectors, (b) the unconditional distribution of **X**_*t*_ is unimodal, and (c) **X**_*t*_ is *S*-mixing.

Assumption **G1**(a) is standard in time series analysis and **G1**(b) is due to [19]. Both relate to the unconditional distribution of **X**_*t*_. Assumption **G1**(c) relates to the conditional distribution and it specifies the time dependence of **X**_*t*_. The purpose of unimodality is twofold. First, it rules out distributions with several modes (but not asymmetry). Second, we consider the Gaussian and uncorrelated (hence independent) process as the benchmark. Regarding **G1**(c), assuming a mixing condition instead of a particular type of dynamic model makes TailCoR applicable to a wide array of processes. The conditions for *S*-mixing, introduced by [20], apply to a large number of processes often used in economics and finance, including GARCH models and its extensions, linear processes (like ARMA models), and stochastic volatility among others.

**Fig 1:**
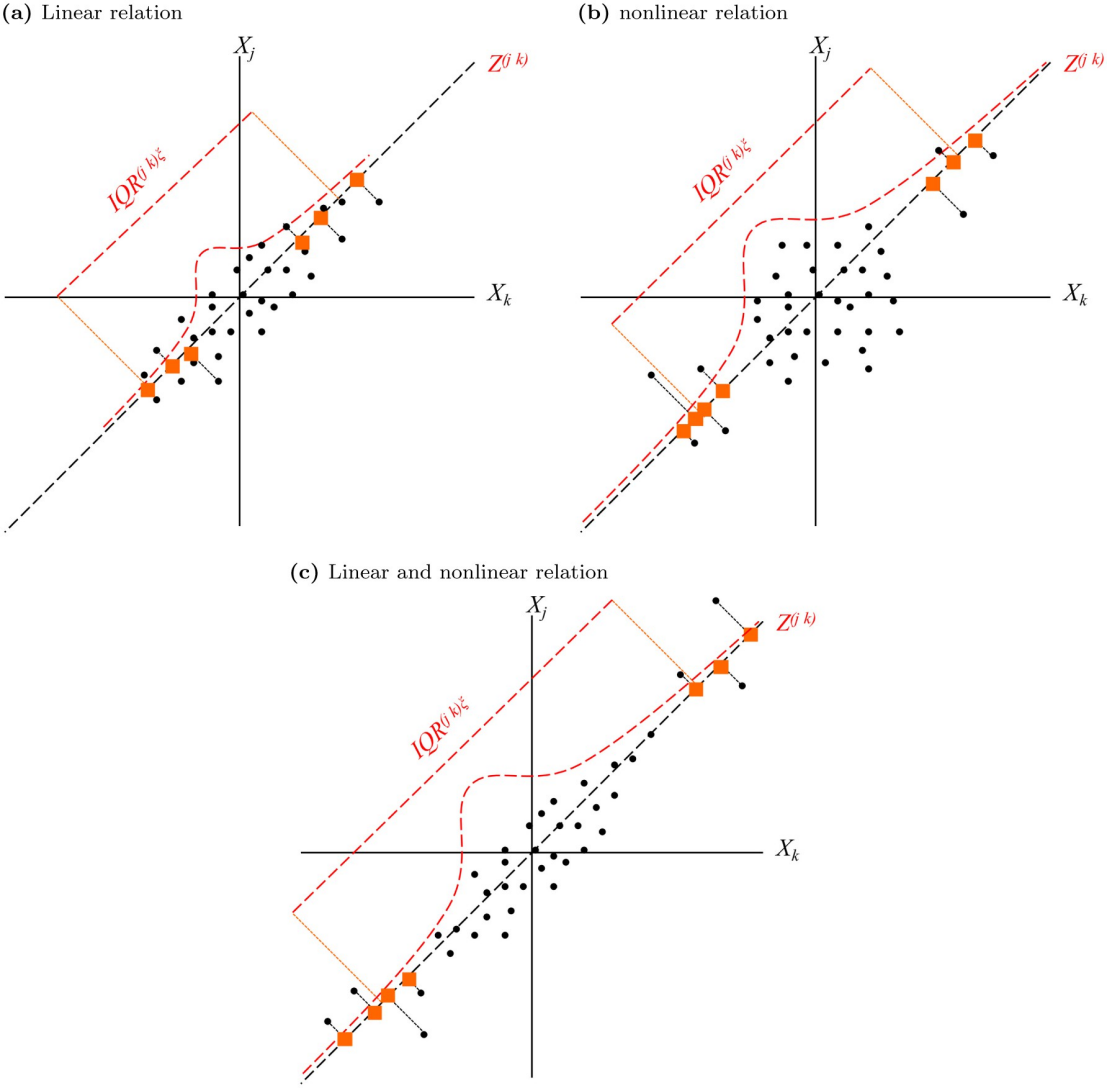
Diagrammatic representation of TailCoR. Scatter plots where *X*_*j*_ and *X*_*k*_ are positively related (the pairs are depicted with circles). Projecting the observations onto the *ϕ*- degree line produces the random variable *Z*^(*jk*)^, depicted with squares. Because of representation purposes we show the projection only for the observations on the tails but the reader should keep in mind that the projection is done for all the observations. Panel (a) shows the case of linear relation only. Panel (b) displays the case of nonlinear relation only. Panel (c) shows the scenario of both linear and nonlinear relations.

TailCoR is based on the following simple idea, shown in Fig 1. If two random variables *X*_*j*_ and *X*_*k*_ (properly standardized) are positively related (either linear and/or nonlinearly), most of the times the pairs of observations (depicted with circles) have the same sign, in the sense that most of them concentrate in the north-east and south-west quadrants of the scatter plot. Now, consider the *ϕ*-degree line that crosses these quadrants (we illustrate the figure with *ϕ* = *π*/4 or the 45-degree line) and project all the pairs on this line, producing a new random variable *Z*^(*jk*)^, depicted with squares (because of representation purposes we show the projection only for the observations that are far from the origin, but the reader should keep in mind that the projection is done for all the observations). Since the two random variables are positively related, the squares—that are sitting on the *ϕ*-degree line—are dispersed all over the line. In the case of negative relation, the dots mostly concentrate in the north-west and south-east quadrants, and the projection is on the corresponding *ϕ*-degree line, as explained in detail later. The extend of the dispersion depends on the strength of the relation between *X*_*j*_ and *X*_*k*_. If weak, the cloud of dots is concentrated around the origin without a well defined direction. The dispersion of the squares is therefore small. By contrast, if the relation is strong, the cloud of dots is stretched around the *ϕ*-degree line, and hence the squares are very dispersed.

TailCoR is equal—up to a normalization—to the difference between the upper and lower tail quantiles of *Z*^(*jk*)^. This *tail interquantile range* can be large because of two reasons. First, if *X*_*j*_ and *X*_*k*_ are highly linearly correlated (panel (a) of Fig 1). Second, if the correlation between *X*_*j*_ and *X*_*k*_ only happens on the tails, while the observations around the origin form a cloud with undefined direction (panel b). These two situations are not mutually exclusive and both may happen, which is actually the most likely case in practice (panel c). Either way, TailCoR is large, in a sense to be precisely defined below. But first we formalize the intuition.

Let *X*_*jt*_ be the *j*th element of the random vector **X**_*t*_. Denote by ${\text{Q}}_{j}^{\tau }$ its *τ*th quantile for 0 < *τ* < 1, and let ${\text{IQR}}_{j}^{\tau }={\text{Q}}_{j}^{\tau }-{\text{Q}}_{j}^{1-\tau }$ be the *τ*th interquantile range. A typical value of *τ* is 0.75. Let *Y*_*jt*_ be the standardized version of *X*_*jt*_:

$$\begin{array}{c} Y_{j\;t}=\frac{X_{j\;t}-{\text{Q}}_{j}^{0.50}}{{\text{IQR}}_{j}^{\tau }}. \end{array}$$ (1)

Likewise for *Y*_*kt*_. In our context of potential heavy tails, the median and the interquantile range are used for the standardization. The mean of *Y*_*jt*_ is not necessarily zero and its variance is not one, if they exist. This is not an issue since the aim of (1) is to have the pair (*Y*_*jt*_, *Y*_*kt*_) centered around the same number and with the same scale. As (1) is based on marginal quantiles, we need the following technical assumption

**G2** The random variable *X*_*jt*_ has *F*(*x*_*j*_) as cumulative distribution function with density *f*(*x*_*j*_) that is continuous and non-zero in a neighbourhood of ${\text{Q}}_{j}^{\tau }$ for *τ* ∈ [0, 1]. Likewise for *X*_*kt*_.

Alternatively to (1), *X*_*jt*_ and *X*_*kt*_ could be standardized with the marginal cumulative distribution functions, i.e. *Y*_*jt*_ and *Y*_*kt*_ would be the probability integral transforms that are distributed uniformly on (0, 1). This is advantageous if there are marginal dependencies beyond the location and the scale, or if we extend our method to copulas (as explained in the conclusions).

By standard trigonometric arguments, the projection of (*Y*_*jt*_, *Y*_*kt*_) on the *ϕ*-degree line is

$$\begin{array}{c} Z_{t}^{\left(j\;k\right)}=Y_{j\;t}\;\text{cos}\;\phi +Y_{k\;t}\;\text{sin}\;\phi , \end{array}$$ (2)

and the tail interquantile range of $Z_{t}^{\left(j\;k\right)}$ is

$$\begin{array}{c} {\text{IQR}}^{(j\;k)\;\xi }={\text{Q}}^{(j\;k)\;\xi }-{\text{Q}}^{(j\;k)\;1-\xi }, \end{array}$$

where *Q*^(*jk*)*ξ*^ is the *ξ*th quantile of $Z_{t}^{\left(j\;k\right)}$ and *ξ* is typically beyond 0.90. The larger *ξ* is, the further we explore the tails. We define TailCoR as follows.

**Definition 1** Under **G1**–**G2**, TailCoR between *X*_*jt*_ and *X*_*kt*_ is

$$\begin{array}{c} {\text{TailCoR}}^{(j\;k)\;\xi }≔s_{0}\left(\xi ,\tau \right){\text{IQR}}^{(j\;k)\;\xi }, \end{array}$$ (3)

where *s*_0_(*ξ*, *τ*) is a normalization such that under independence TailCoR^(*jk*)*ξ*^ = 1, the reference value.

Four remarks are in order. First, the meaning of correlation in TailCoR is not the traditional one, as TailCoR is not bounded between -1 and 1, nor centered at zero. Second, *s*_0_(*ξ*, *τ*) equals the inverse of the *IQR*^(*jk*)*ξ*^ under independence. In the next sub-section, we show that in the case of ellipticity, *s*_0_(*ξ*, *τ*) can be computed from a standard Gaussian distribution.

Third, TailCoR is not an asymptotic (in the tail) dependence measure. Instead, the dependence between *X*_*jt*_ and *X*_*kt*_ can be computed precisely for any *ξ*, and, since TailCoR does not rely on assumptions of the asymptotic behavior of the tail, it can be used for distributions with tails that are Pareto, exponential, or even with finite end points.

Last, the angle *ϕ* has to be chosen. An optimality criterion would be to choose the angle between 0 and *π* that maximizes the *IQR*^(*jk*)*ξ*^. In practice this can be done with a grid search on *ϕ*, computing the projection and the tail interquantile range for each angle of the grid. Since no optimizations are involved, this grid search is computationally inexpensive.

### 2.2 Disentangling the linear and nonlinear components

#### Ellipticity and assumptions

Though definition (3) is simple and intuitive, it does not allow to understand how much of TailCoR is due to the linear and nonlinear dependencies. This is possible however if we assume ellipticity.

**E1** The unconditional distribution of **X**_*t*_ belongs to the elliptical family, given by the stochastic representation $X_{t}{=}_{d}\mu +R_{\alpha \;t}\Lambda U_{t}$.

The *N* × 1 random vector **U**_*t*_ under **E1** is uniformly distributed on the unit sphere. The scaling matrix **Λ** produces the ellipticity and is such that **Σ** = **ΛΛ**′, a positive definite symmetric dispersion matrix—often called the shape matrix with generic (*jk*) element $\sigma_{X_{j}\;X_{k}}$, and for convenience we denote the (*jj*) diagonal element by $\sigma_{X_{j}}^{2}$. The non-negative and continuous random variable $R_{\alpha \;t}$ generates the tail thickness through a so-called radial density depending on the shape parameter *α*, and is stochastically independent of **U**_*t*_. From now on, we will denote *α* as the tail index, in the sense that this parameter explains the decay of the tails (the smaller *α* the thicker the tails), but not necessarily according to a power law. The random variable $R_{\alpha \;t}$ may not only depend on the tail index but on a vector of shape parameters. We do not consider the latter since most of the elliptical distributions used in practice only depend on *α* (see examples after assumption **E2**). The extension is however straightforward. The vector ***μ*** re-allocates the center of the distribution. Let ***θ*** = (***μ***, **Σ**, *α*) ∈ **Θ** denote the vector of unknown parameters satisfying the following standard assumption.

**E2** (a) The parameter space **Θ** is a non-empty and compact set on $R^{N+\frac{N(N+1)}{2}+1}$. (b) The true parameter value ***θ***_0_ belongs to the interior of **Θ**.

Note that assumption **E1** implies that the unconditional distribution of **X**_*t*_ belongs to the elliptical family, but no specific distributional assumption is made. The elliptical family nests, among others, the Gaussian, Student-*t*, elliptical stable (ES henceforth), Cauchy, Laplace, power exponential, and Kotz probability laws; see [21, 22] for tail theory within the elliptical family. For a given vector of locations and a dispersion matrix, the difference between two elliptical distributions is the random quantity $R_{\alpha \;t}$ with tail index *α*, which plays a central role here. Another feature of the elliptical family is its closeness under location and scale shifts, which implies that $Z_{t}^{\left(j\;k\right)}$ is elliptical with the same tail index *α*.

We need the existence of the mean and the variance-covariance matrix:

**E3** The unconditional moments up to order 2 are finite, i.e. $E(X_{t}^{p})<\infty ,\;\text{for}\;p\leq 2$.

Next, we substitute assumptions **G1** and **G2** for

**E4** (a) The random process {**X**_*t*_} is a weakly stationary sequence of random vectors, and (b) **X**_*t*_ is *S*-mixing.
**E5** The random variable $R_{\alpha \;t}$ has *P*(*r*) as cumulative distribution function with density *p*(*r*) that is continuous and non-zero in a neighbourhood of ${\text{Q}}_{j}^{\tau }$ for *τ* ∈ [0, 1].

#### The optimal projection

Ellipticity allows to compute the optimal angle *ϕ* without the grid search mentioned above. First, note that the interquantile range used in the standardization (1) can be written as $k\left(\tau ,\alpha \right)\sigma_{X_{j}}$, where *k*(*τ*, *α*) is a non-random positive constant. Likewise ${\text{IQR}}_{k}^{\tau }=k\left(\tau ,\alpha \right)\sigma_{X_{k}}$ ([23] introduced this relation between the IQR and the scale parameter in the context of stable distributions). Then, by **E1** the bivariate random vector $Y_{t}^{j\;k}=\left(Y_{j\;t},Y_{k\;t}\right)$ is elliptically distributed with a 2 × 2 shape matrix *k*(*τ*, *α*)^−2^**R**. The matrix **R** has diagonal elements *ρ*_11_ = *ρ*_22_ = 1 and off diagonal element $\rho_{12}=\sigma_{X_{j}\;X_{k}}/\sigma_{X_{j}}\sigma_{X_{k}}$ (3.3.2 of [6]).

Therefore, the probability contours of *Y*^*jk*^ are ellipsoids with axes that are in the direction of the eigenvectors of *k*(*τ*, *α*)^2^**R**^−1^, and their lengths are proportional to the reciprocals of the square roots of the eigenvalues of *k*(*τ*, *α*)^2^**R**^−1^. Since *ρ*_11_ = *ρ*_22_ = 1, the first eigenvalue is *k*(*τ*, *α*)^−2^(1 + *ρ*_12_) with associated eigenvector $\left(1/\sqrt{2},1/\sqrt{2}\right)$, while the second eigenvalue is *k*(*τ*, *α*)^−2^(1 − *ρ*_12_) with associated eigenvector $\left(1/\sqrt{2},-1/\sqrt{2}\right)$.

If the relation between *X*_*jt*_ and *X*_*kt*_ is positive, then *ρ*_12_ is positive and *k*(*τ*, *α*)^−2^(1 + *ρ*_12_) is the largest eigenvalue. Hence the projection on the 45-degree line is optimal:

$$\begin{array}{c} Z_{t}^{\left(j\;k\right)}=\frac{1}{\sqrt{2}}\left(Y_{j\;t}+Y_{k\;t}\right). \end{array}$$ (4)

If *ρ*_12_ is negative, then *k*(*τ*, *α*)^−2^(1 − *ρ*_12_) is the largest eigenvalue and the projection in the 135-degree line is optimal:

$$\begin{array}{c} Z_{t}^{\left(j\;k\right)}=\frac{1}{\sqrt{2}}\left(Y_{j\;t}-Y_{k\;t}\right). \end{array}$$ (5)

**Fig 2:**
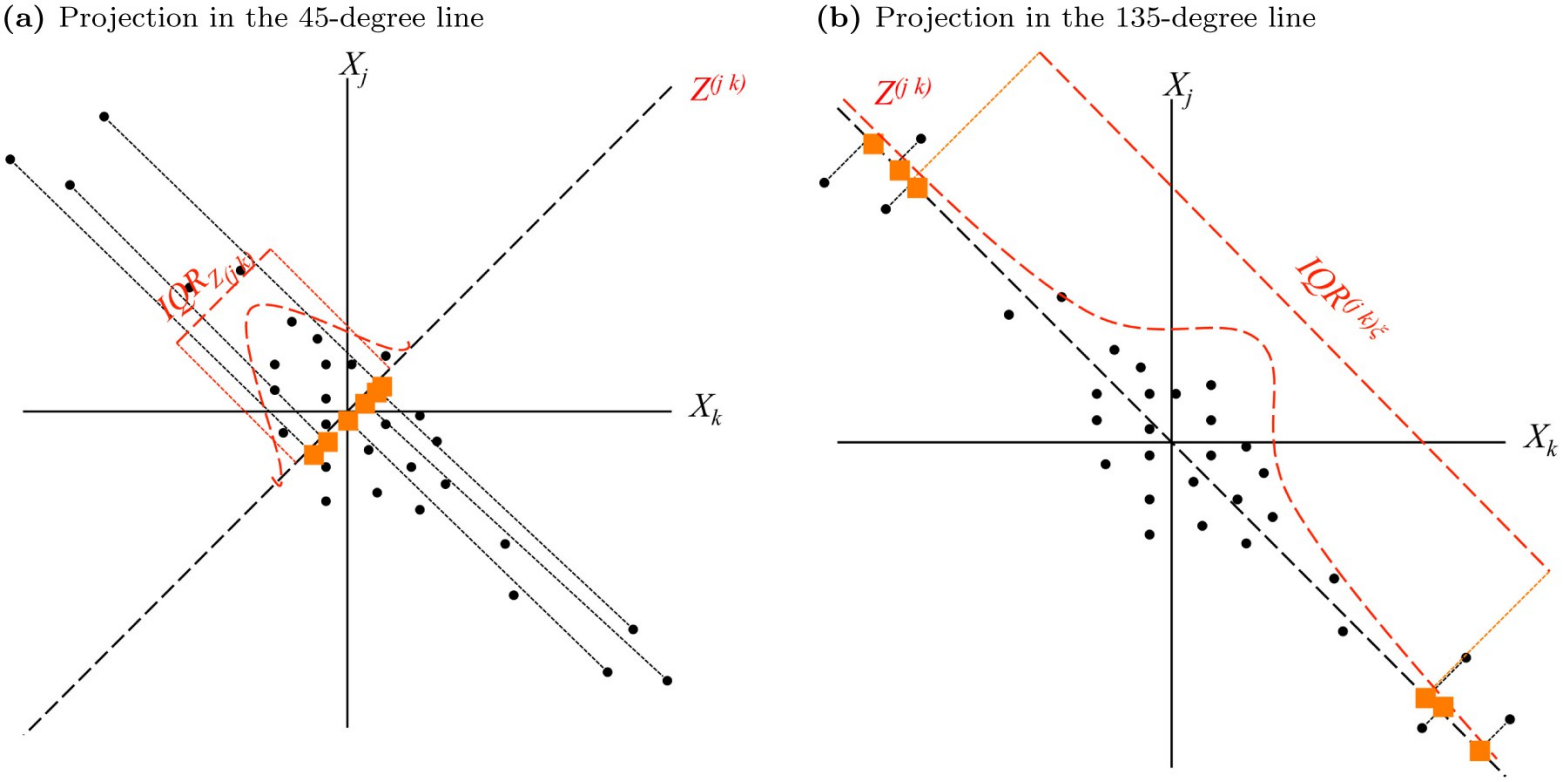
A diagrammatic representation of TailCoR for negative relation. Scatter plots where *X*_*j*_ and *X*_*k*_ are negatively related and the projection is done with the wrong and right angles (panels (a) and (b) respectively).

Either way, $Z_{t}^{\left(j\;k\right)}$ is the first principal component of *Y*^*jk*^. The choice between the 45- and the 135-degree lines depends on the sign of *ρ*_12_. If wrongly chosen, conclusions may be misleading, as exemplified in Fig 2. Panel (a) shows the projection on the 45-degree line when the relation is negative. The projection *Z*^(*jk*)^ is concentrated around the origin and TailCoR^(*jk*)*ξ*^ can even be smaller than one, leading to the false conclusion that tails are thinner than Gaussian. Projecting on the 135-degree line, as shown in panel (b), captures correctly the negative relation between *X*_*jt*_ and *X*_*kt*_.

#### TailCoR under ellipticity

We are now ready to disentangle TailCoR^(*jk*)*ξ*^ into the linear and nonlinear components.

**Theorem 1** Let *X*_*jt*_ and *X*_*kt*_ be two elements of the random vector **X**_*t*_ that fulfills assumptions **E1**–**E5**. Let *ρ*_*jk*_ be the linear correlation, and let *s*(*ξ*, *τ*, *α*) be a continuous and monotonically decreasing function of *α*. Then

$$\begin{array}{c} {\text{TailCoR}}^{(j\;k)\;\xi }=s_{g}\left(\xi ,\tau \right)s\left(\xi ,\tau ,\alpha \right)\sqrt{1+|\rho_{j\;k}|}, \end{array}$$

where *s*_*g*_(*ξ*, *τ*) is a normalization such that under independence TailCoR^(*jk*)*ξ*^ = 1, the reference value.

**Proof** See S1 Appendix.

The rightmost element, $\sqrt{1+|\rho_{j\;k}|}$, captures the linear dependency of TailCoR^(*jk*)*ξ*^, while *s*(*ξ*, *τ*, *α*) captures the nonlinear dependency as it depends on the tail index *α*. We denote these dependencies as the linear and nonlinear components.

The normalization has become now *s*_*g*_(*ξ*, *τ*) where the subindex *g* is for Gaussianity. Indeed, under ellipticity, independence translates into Gaussianity and linear uncorrelation and hence $s_{g}\left(\xi ,\tau \right)=\frac{\Phi^{-1}\left(\tau \right)}{\Phi^{-1}\left(\xi \right)}$ where Φ(⋅) is the cumulative distribution function of a standardized Gaussian distribution. This can be shown using results of Theorem 1. Under Gaussianity and linear uncorrelation, $Z_{t}^{\left(j\;k\right)}∼\frac{1}{2\Phi^{-1}\left(\tau \right)}N\left(0,1\right)$. Hence ${\text{IQR}}^{(j\;k)\;\xi }=\frac{\Phi^{-1}\left(\xi \right)}{\Phi^{-1}\left(\tau \right)}$. Since *s*_*g*_(*ξ*, *τ*)IQR^(*jk*)*ξ*^ = 1, then $s_{g}\left(\xi ,\tau \right)=\frac{\Phi^{-1}\left(\tau \right)}{\Phi^{-1}\left(\xi \right)}$. Values of *s*_*g*_(*ξ*, *τ*) can be easily programmed, or one can look into the first table of the separate S2 Appendix which shows values of *s*_*g*_(*ξ*, *τ*) for a grid of reasonable values for *τ* and *ξ*.

**Fig 3:**
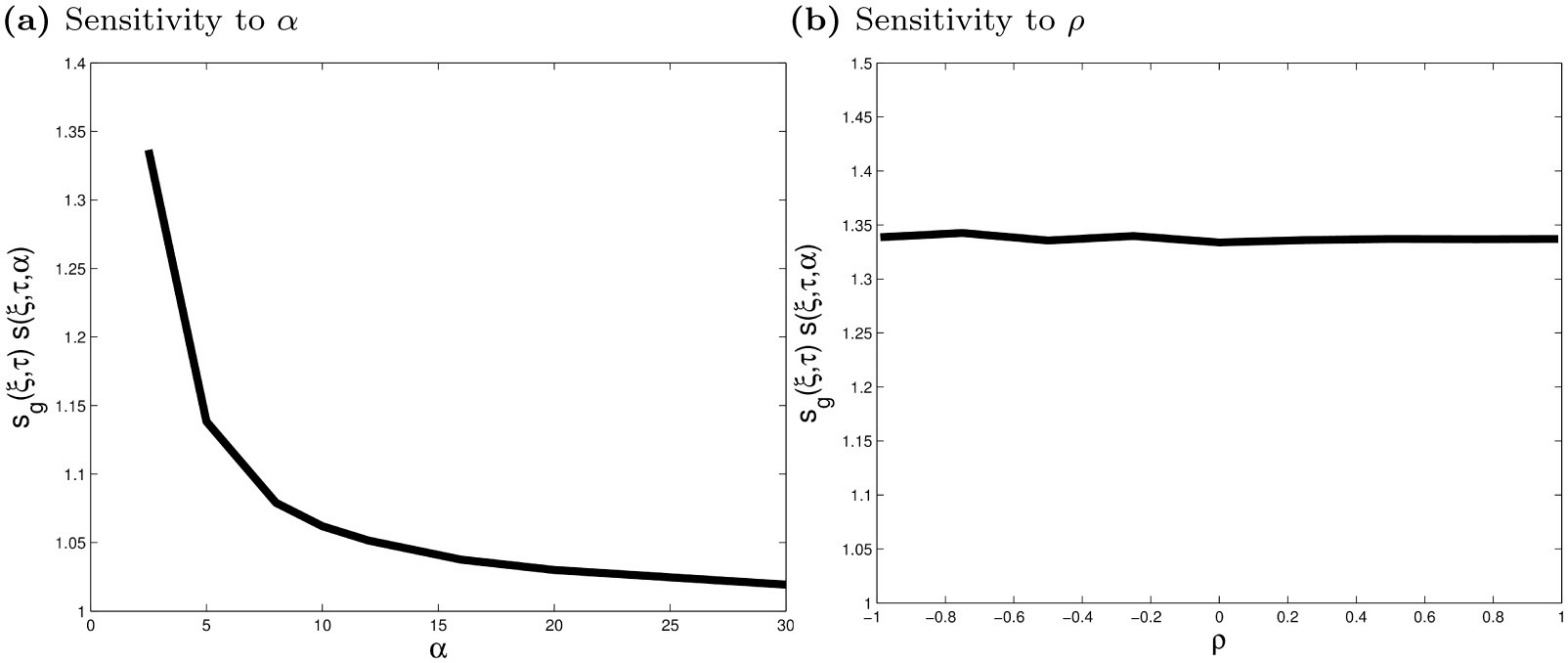
Sensitivity of *s*_*g*_(*ξ*, *τ*)*s*(*ξ*, *τ*, *α*) to *α* and *ρ*. Panel (a) shows the sensitivity of the nonlinear component to *α*. The tail index varies from 2.5 to 30. Panel (b) shows the sensitivity to *ρ* (for *α* = 2.5). Both plots are for *τ* = 0.75 and *ξ* = 0.95.

Panel (a) of Fig 3 displays *s*_*g*_(*ξ*, *τ*)*s*(*ξ*, *τ*, *α*) as a function of *α* and assuming a Student-*t* distribution (and for *ξ* = 0.95 and *τ* = 0.75). The tail index varies from 2.5 to 30. The nonlinear component decreases as *α* increases, and *s*_*g*_(*ξ*, *τ*)*s*(*ξ*, *τ*, *α*) approaches 1 as *α* goes to 30, or when the distribution is indistinguishable from the Gaussian. Panel (b) shows the sensitivity of *s*_*g*_(*ξ*, *τ*)*s*(*ξ*, *τ*, *α*) to *ρ* (with *α* = 2.5). The nonlinear component is not affected by *ρ*, which confirms that $\sqrt{1+|\rho_{j\;k}|}$ and *s*(*ξ*, *τ*, *α*) capture different aspects of TailCoR^(*jk*)*ξ*^.

TailCoR^(*jk*)*ξ*^ has numerous properties. First, it captures nonlinear dependencies if tails deviate from Gaussianity. That is *s*(*ξ*, *τ*, *α*)*s*_*g*_(*ξ*, *τ*) can be greater, equal, or smaller than 1, which corresponds to tails fatter, equal, or thinner than the Gaussian distribution. Second, even if *X*_*j*_ and *X*_*k*_ are linearly uncorrelated, TailCoR^(*jk*)*ξ*^ is different from 1 if *X*_*j*_ and *X*_*k*_ are non-Gaussian. This is akin to the coefficient of tail dependence stemming from copula theory. For instance, the tail dependence coefficient of a bivariate Student-*t* copula is $2t_{\alpha +1}(-\sqrt{\frac{\left(\alpha +1)(1-\rho \right)}{1+\rho }})$, where *t*_*α*+1_(⋅) is a standardized Student-*t* cumulative distribution function with tail index *α* + 1. Even if *ρ* = 0 tail dependence is positive (unless *α* → ∞). Third, if **X**_*t*_ is Gaussian, *s*(*ξ*, *τ*, *α*)*s*_*g*_(*ξ*, *τ*) = 1 and ${\text{TailCoR}}^{(j\;k)\;\xi }=\sqrt{1+|\rho_{j\;k}|}$, i.e. the only source of dependence is linear.

**Fig 4:**
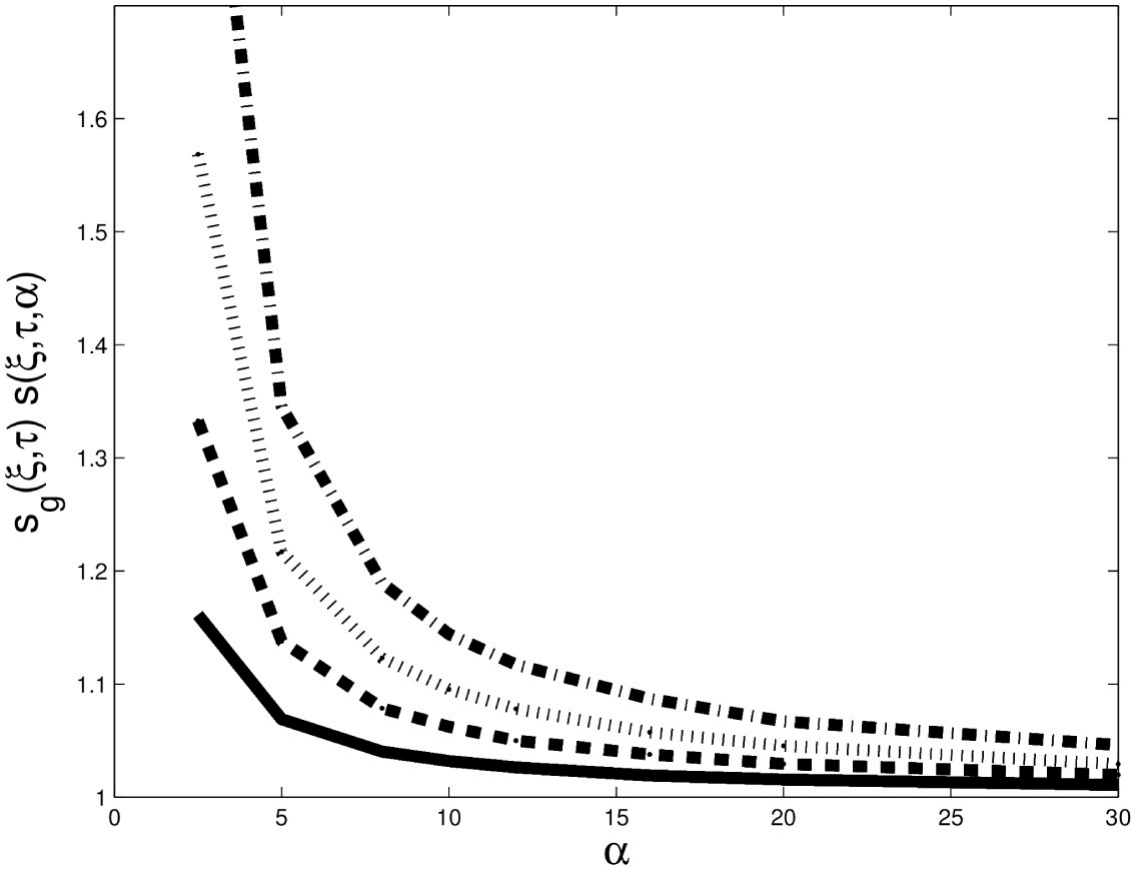
Sensitivity of *s*_*g*_(*ξ*, *τ*)*s*(*ξ*, *τ*, *α*) to *α* and *ξ*. Sensitivity of the nonlinear correlation to *α* for the Student-*t* distribution. The tail index varies from 2.5 to 30. Each line is for a value of *ξ*: 0.90 (solid line), 0.95 (thick dashes), 0.975 (thin dashes) and 0.99 (thick and thin dashes).

This last property shows that under Gaussianity the upper bound is $\sqrt{2}$. Otherwise, the upper bound depends on *α* and *ξ*. Fig 4 displays similar curves to those in panel (a) of Fig 3 for values of *ξ* typically used in practice: 0.90 (solid line), 0.95 (thick dashes), 0.975 (thin dashes), and 0.99 (thick and thin dashes). The further we explore the tails, the larger *s*_*g*_(*ξ*, *τ*)*s*(*ξ*, *τ*, *α*) is, and so TailCoR is. The range of values is 1 − 1.5 (except if *ξ* is close to 1 and tails are very heavy), which in turn translates into a range of values of TailCoR between 1 and 2.12 (e.g., if *ρ* = 1, TailCoR equals $\sqrt{2}\times 1.5=2.12$).

### 2.3 Estimation

Estimation under **G1**–**G2** is straightforward and divided in three simple steps.

**Step 1** Standardize *X*_*jt*_ with the sample median ${\hat{\text{Q}}}_{j}^{0.50}$ and the sample interquantile range ${\hat{\text{IQR}}}_{j}^{\tau }.$ Likewise for *X*_*kt*_. Then compute the projection $Z_{t}^{\left(j\;k\right)}$.
**Step 2** Estimate the IQR of the projection: ${\hat{\text{IQR}}}_{T}^{(j\;k)\;\xi }$.
**Step 3** Compute the normalization *s*_*g*_(*ξ*, *τ*) and ${\hat{\text{TailCoR}}}_{T}^{(j\;k)\;\xi }\left({\hat{\text{Q}}}_{j}^{0.50},{\hat{\text{Q}}}_{k}^{0.50},{\hat{\text{IQR}}}_{j}^{\tau },{\hat{\text{IQR}}}_{k}^{\tau }\right)=s_{g}\left(\xi ,\tau \right){\hat{\text{IQR}}}_{T}^{(j\;k)\;\xi }$. This somehow cumbersome notation emphasises that TailCoR is a function of estimated quantiles (step 1).

Under **E1**–**E5** the linear correlation *ρ*_*jk*_ is estimated with a robust method. [24] introduce a robust estimator that is invariant in the class of elliptical distributions. Let ${\hat{\kappa }}_{j\;k,T}$ be the estimator of Kendall’s correlation. Then

$$\begin{array}{c} {\hat{\rho }}_{j\;k,T}=\text{sin}(\frac{\pi }{2}{\hat{\kappa }}_{j\;k,T}), \end{array}$$

and $\sqrt{1+|{\hat{\rho }}_{j\;k,T}|}$ follows. Given the ${\hat{\text{IQR}}}_{T}^{(j\;k)\;\xi }$ obtained in step 2 above, the estimator of the nonlinear component is

$$\begin{array}{c} \hat{s}{\left(\xi ,\tau ,\alpha \right)}_{T}=\frac{{\hat{\text{IQR}}}_{T}^{(j\;k)\;\xi }}{\sqrt{1+|{\hat{\rho }}_{j\;k,T}|}}. \end{array}$$

We now see the computational advantages of TailCoR^(*jk*)*ξ*^. It can be estimated exactly for any probability level *ξ* and no optimizations are needed, as it is based on simple steps each requiring a few lines of programming code. This makes TailCoR^(*jk*)*ξ*^ fast to compute. Moreover, estimation of the tail index is not required.

The following theorem shows the consistency of ${\hat{\text{TailCoR}}}_{T}^{(j\;k)\;\xi }$.

**Theorem 2** Let *X*_*jt*_ and *X*_*kt*_ be two elements of the random vector **X**_*t*_ that fulfills assumptions **G1**–**G2**. Then, as *T* → ∞

$$\begin{array}{c} {\hat{\text{TailCoR}}}_{T}^{(j\;k)\;\xi }\left({\hat{\text{Q}}}_{j}^{0.50},{\hat{\text{Q}}}_{k}^{0.50},{\hat{\text{IQR}}}_{j}^{\tau },{\hat{\text{IQR}}}_{k}^{\tau }\right)-{\text{TailCoR}}^{(j\;k)\;\xi }=o_{p}\left(1\right). \end{array}$$

**Proof** See S1 Appendix.

Next, we prove asymptotic normality. Admittedly, the theorem ignores the effect of the estimated median and the IQR in the standardization of *X*_*jt*_ and *X*_*kt*_ (step 1). Monte Carlo results shown below indicate that the effect of step 1 is negligible.

**Theorem 3** Let *X*_*jt*_ and *X*_*kt*_ be two elements of the random vector **X**_*t*_ that fulfills assumptions **E1**–**E5**. Let *f*_(*jk*)_(⋅) and *F*_(*jk*)_(⋅) be the probability density and cumulative distribution functions of $Z_{t}^{\left(j\;k\right)}$, and let *I*_{⋅}_ be an indicator function that takes value 1 if its argument is true. Then, as *T* → ∞

$$\begin{array}{c} \sqrt{T}({\hat{\text{TailCoR}}}_{T}^{(j\;k)\;\xi }\left({\text{Q}}_{j}^{0.50},{\text{Q}}_{k}^{0.50},{\text{IQR}}_{j}^{\tau },{\text{IQR}}_{k}^{\tau }\right)-{\text{TailCoR}}^{(j\;k)\;\xi })\to_{d}N(0,4s_{g}{\left(\xi ,\tau \right)}^{2}\frac{\Gamma (Q^{(j\;k)\xi })}{f_{\left(j\;k\right)}^{2}\left(F_{\left(j\;k\right)}^{-1}\left(\xi \right)\right)}), \end{array}$$

where

$$\begin{array}{ccc} \Gamma (Q^{(j\;k)\xi }) & = & \sum_{t=-\infty }^{+\infty }E(W_{0}\left(Q^{(j\;k)\xi }\right)W_{t}\left(Q^{(j\;k)\xi }\right)),\\ W_{t}\left(Q^{(j\;k)\xi }\right) & = & I_{\left\{Z_{t}^{\left(j\;k\right)}\leq Q^{(j\;k)\xi }\right\}}-P\left(Z_{t}^{\left(j\;k\right)}\leq Q^{(j\;k)\xi }\right), \end{array}$$

and →_*d*_ stands for convergence in distribution.

**Proof** See S1 Appendix.

We make the following remarks about the theorem. First, the univariate density *f*_(*jk*)_(⋅) in the denominator is symmetric, and therefore easy to compute. Second, Γ(*Q*^(*jk*)*ξ*^) is the long-run component of the variance that accounts for the time dependence. Third, the asymptotic variance can be computed by bootstrap, as we do in the empirical application (we use block bootstrap of length 50 and 500 replications). Last, it is possible to derive an equivalent asymptotic distribution under **G1**–**G2**, as shown in the following corollary.

**Corollary 1** Let *X*_*jt*_ and *X*_*kt*_ be two elements of the random vector **X**_*t*_ that fulfills assumptions **G1**–**G2**. Let *f*_(*jk*)_(⋅) and *F*_(*jk*)_(⋅) be the probability density and cumulative distribution functions of $Z_{t}^{\left(j\;k\right)}$, and let *I*_{⋅}_ be an indicator function that takes value 1 if its argument is true. Then, as *T* → ∞

$$\begin{array}{c} \sqrt{T}({\hat{\text{TailCoR}}}_{T}^{(j\;k)\;\xi }\left({\text{Q}}_{j}^{0.50},{\text{Q}}_{k}^{0.50},{\text{IQR}}_{j}^{\tau },{\text{IQR}}_{k}^{\tau }\right)-{\text{TailCoR}}^{(j\;k)\;\xi })\to_{d}N(0,s_{g}{\left(\xi ,\tau \right)}^{2}ϒ), \end{array}$$

where

$$\begin{array}{c} ϒ=\frac{\Gamma (Q^{(j\;k)\xi })}{f_{\left(j\;k\right)}^{2}\left(F_{\left(j\;k\right)}^{-1}\left(\xi \right)\right)}+\frac{\Gamma (Q^{(j\;k)1-\xi })}{f_{\left(j\;k\right)}^{2}\left(F_{\left(j\;k\right)}^{-1}\left(1-\xi \right)\right)}-2\frac{\Gamma (Q^{(j\;k)\xi },Q^{(j\;k)1-\xi })}{f_{\left(j\;k\right)}\left(F_{\left(j\;k\right)}^{-1}\left(\xi \right)\right)f_{\left(j\;k\right)}\left(F_{\left(j\;k\right)}^{-1}\left(1-\xi \right)\right)}, \end{array}$$

$$\begin{array}{c} \Gamma \left(Q^{(j\;k)\xi },Q^{(j\;k)1-\xi }\right)=\sum_{t=-\infty }^{+\infty }E(W_{0}\left(Q^{(j\;k)\xi }\right)W_{t}\left(Q^{(j\;k)1-\xi }\right)),\;\text{and}\; \end{array}$$

$$\begin{array}{c} W_{t}\left(Q^{(j\;k)\xi }\right)=I_{\left\{Z_{t}^{\left(j\;k\right)}\leq Q^{(j\;k)\xi }\right\}}-P\left(Z_{t}^{\left(j\;k\right)}\leq Q^{(j\;k)\xi }\right). \end{array}$$

**Proof** If follows from the proof of Theorem 3 but with *Q*^(*jk*)*ξ*^ ≠ −*Q*^(*jk*)1−*ξ*^.

## 3 A monte carlo simulation study

We analyze the finite sample properties of TailCoR with three bivariate elliptical distributions: Gaussian, Student-*t* with *α* = 2.5, and ES with *α* = 1.5. The most heavy tailed distribution is the Student-*t*, followed by the ES and the Gaussian. The location parameters are set to zero and the dispersion matrix has unitary diagonal elements and off-diagonal elements 0.50. We consider three sample sizes *T* = {1000, 5000, 10000} and two replication sizes *H* = {1000, 10000}. In the sequel we show results for *T* = 10000 and *H* = 10000, unless otherwise stated. Results for other configurations are alike and they are available upon request.

**Fig 5:**
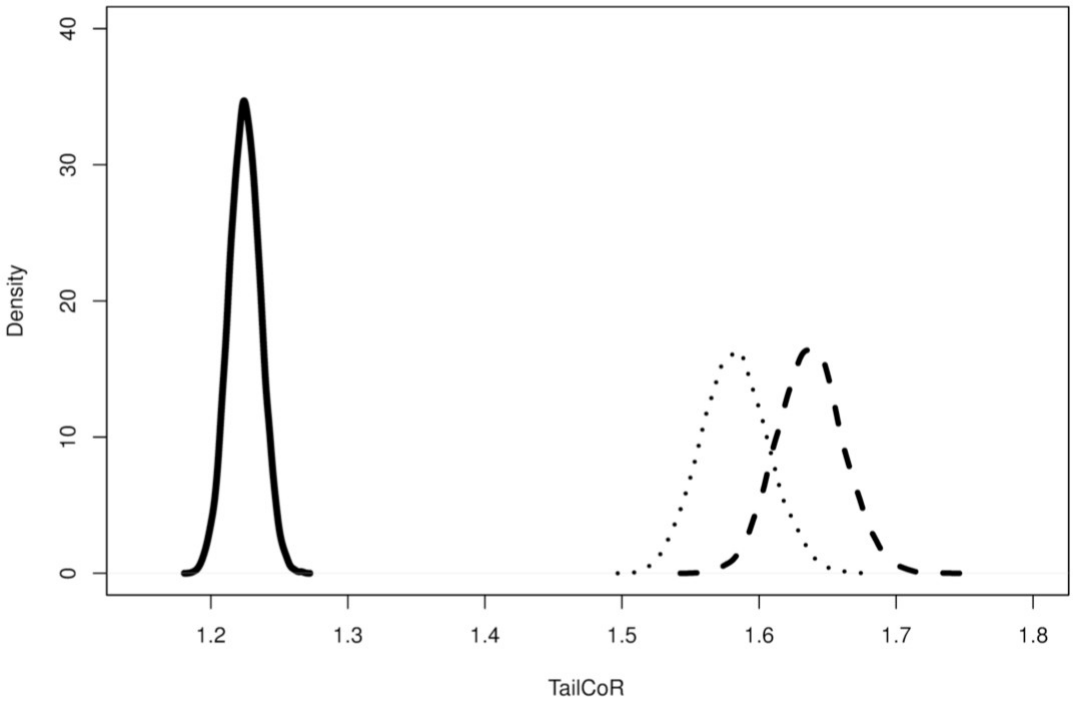
TailCoR for different distributions. Distribution of 10000 estimated TailCoR’s for *ξ* = 0.95 and three distributions: Gaussian (solid line), Student-*t* with *α* = 2.5 (dashed line), ES with *α* = 1.5 (dotted line).

Fig 5 shows the finite sample distributions of the TailCoR estimates for *ξ* = 0.95 and for the three distributions (solid line for the Gaussian, dashed for the Student-*t*, and dotted for the ES). In all cases, TailCoR is larger than one. The estimated TailCoR is more precise under Gaussianity than under heavy tails, as it only depends on the linear correlation. Moreover, the median is around 1.22, very close to the true value $\sqrt{1+0.50}=1.225$. Estimates under the Student-*t* and the ES have higher medians (1.58 for the ES and 1.64 for the Student-*t*), reflecting the nonlinear dependencies.

**Fig 6:**
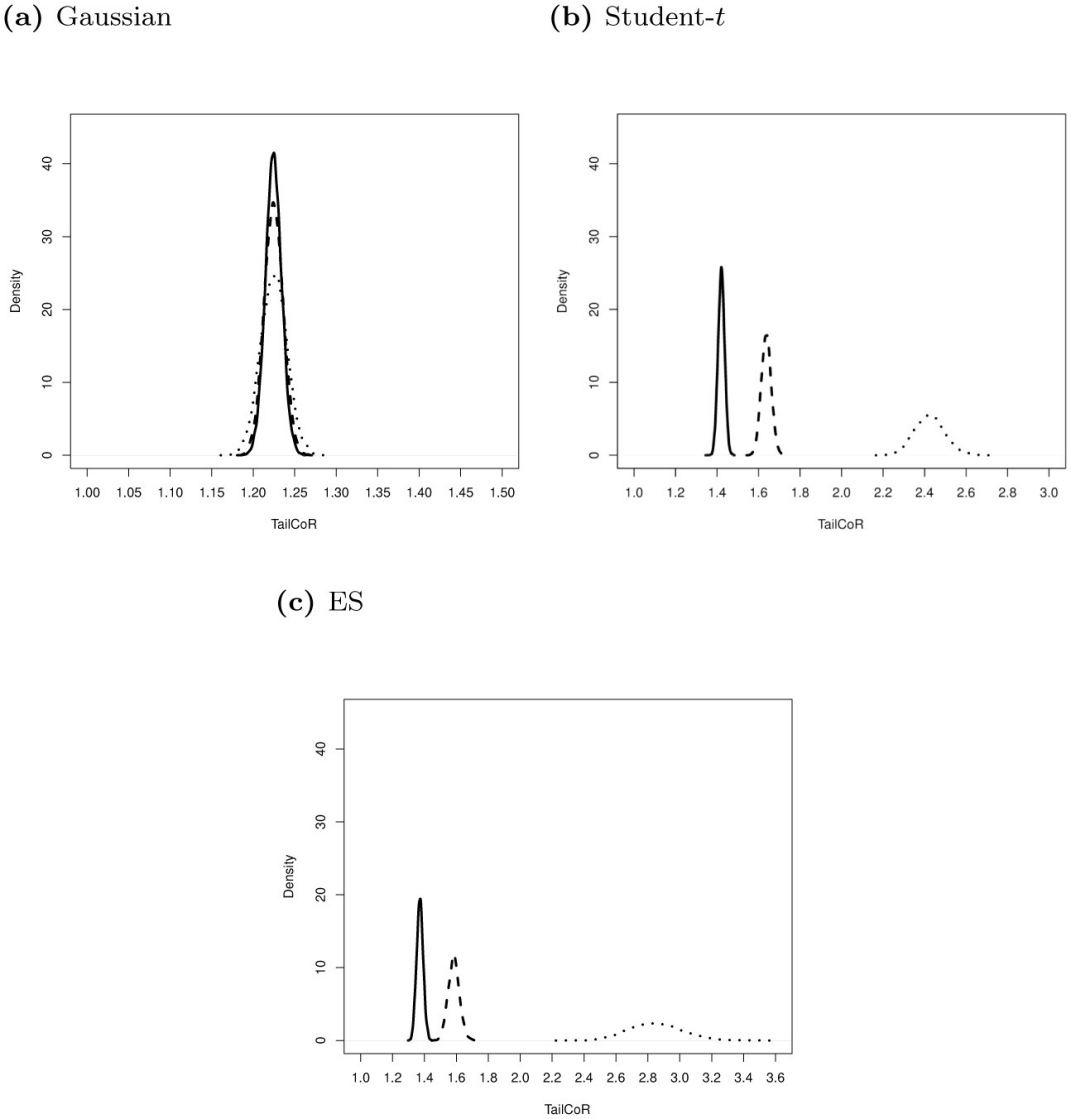
Sensitivity of TailCoR to *ξ*. Sensitivity of TailCoR to *ξ* for the Gaussian (panel a), Student-*t* with *α* = 2.5 (panel b), and ES with *α* = 1.5 (panel c) distributions. Each line is the density of the 10000 estimates of TailCoR for different values of *ξ*: 0.90 (solid line), 0.95 (dashed) and 0.99 (dotted). (a) Gaussian, (b) Student-*t*, (c) ES.

Fig 6 shows the sensitivity of TailCoR to *ξ* for the Gaussian (panel (a)), Student-*t* with *α* = 2.5 (panel (b)), and ES with *α* = 1.5 (panel (c)). Solid lines are for *ξ* = 0.90, dashed for *ξ* = 0.95, and dotted for *ξ* = 0.99. The densities overlap for the Gaussian distribution since TailCoR^(*jk*)*ξ*^ does not depend on *ξ* (it equals $\sqrt{1+|\rho_{j\;k}|}$). The small differences are due to finite sample discrepancies. Regarding the other distributions, results show that, as expected, TailCoR increases with *ξ*.

**Fig 7:**
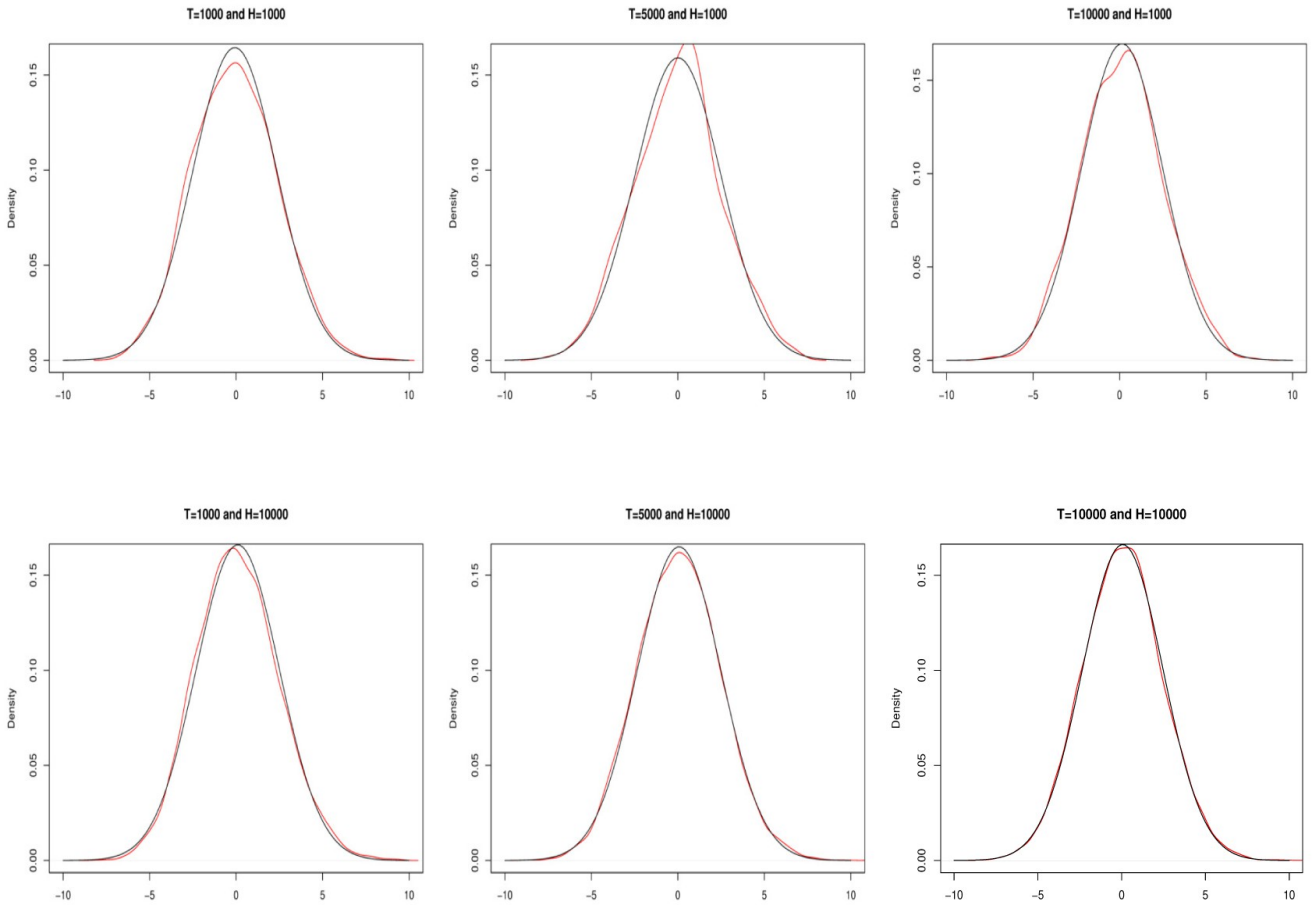
Convergence in distribution. Kernel densities (red line) of the centered estimates against a corresponding Gaussian distribution (black line) for all the combinations of the sample size and replications (indicated in the top of each plot).

The precision and convergence in distribution of the estimator are shown in Fig 7 and in Table 1 for *ξ* = 0.95. Red lines in the figure are the kernel densities of the centered estimates for different sample sizes and replications (indicated at the top of each plot), and the black lines are the corresponding Gaussians. As expected, the kernel densities approach to the limiting distribution as the sample size and the number of replications increase. The table shows the effect of the estimation error of step 1. It compares TailCoR and its components when the population and the sample quantiles are used in step 1 (as shown in the row “Step 1”). Estimations are done for the Gaussian and Student-*t* distributions, two sample sizes (1000 and 10000), 1000 replications, and *ξ* = 0.95. Mean estimates are very close to the true values, indicating unbiasedness. The standard errors are very small and, more importantly, using the sample quantiles in step 1 does not affect significantly the accuracy of the estimates in step 2.

**Table 1:** Estimation uncertainty step 1.

Step 1:		Popul.	Sample	Popul.	Sample
		Gaussian		Student-*t*	
		TailCoR			
*T* = 1000	True	1.225	1.225	1.637	1.637
	Mean	1.223	1.224	1.639	1.635
	SD	0.034	0.037	0.082	0.077
*T* = 10000	Mean	1.225	1.225	1.637	1.637
	SD	0.011	0.011	0.024	0.024
		*s*(*ξ*, *τ*, *α*)			
*T* = 1000	True	2.438	2.438	3.259	3.259
	Mean	2.435	2.438	3.266	3.257
	SD	0.059	0.072	0.152	0.147
*T* = 10000	Mean	2.439	2.438	3.260	3.259
	SD	0.020	0.023	0.045	0.046
		$\sqrt{1+|\rho_{j\;k}|}$			
*T* = 1000	True	1.225	1.225	1.225	1.225
	Mean	1.225	1.224	1.224	1.224
	SD	0.011	0.011	0.012	0.012
*T* = 10000	Mean	1.225	1.225	1.224	1.225
	SD	0.003	0.003	0.004	0.004

Means and standard deviations of estimates of TailCoR and its components when using population (Popul. in top row) or sample marginal quantiles in step 1. All results are for H = 1000 and *ξ* = 0.95

## 4 TailCoR of 21 financial market indexes around the globe

We illustrate TailCoR with an application to 20.5 years of daily stock log returns of 21 major equity market indexes that represent three geographical regions: America (S&P,NASDAQ, TSX, Merval, Bovespa and IPC), Europe (AEX, ATX, FTSE, DAX, CAC40, SMI and MIB), and East Asia and Oceania (HgSg, Nikkei, StrTim, SSEC, BSE, KLSE, KOSPI and AllOrd). The sample spans from 5 January 2000 to 9 July 2020 (each series contains 5369 observations).

Though it is of great interest to apply TailCoR to most recent data, mind that the end of the sample considered here covers the first 6 months of COVID which were the most stressful for global financial markets since the beginning of the pandemic. In March 2020, the VIX (the main index of the risk in US equity markets) skyrocketed to 66%, one of the highest values on record and only surpassed by the peak of the great financial crisis in autumn 2008. After July 2020, VIX decreased to approximately 25% and, with ups and downs, it has remained on this level ever since and till the time of writing these lines in mid-September 2022.

Table 2 shows the countries and descriptive statistics. All medians are approximately zero, as expected. The IQRs are a measure of volatility. If annualised (i.e. multiplied by $\sqrt{252}$), the maximum is Merval with 35% and the minimum is KSLE with 12%. European markets have annualised IQRs between 24% for MIB and 17.8% for SMI, while the IQR for S&P is 16.5%, very close to 15%, the value that market participants consider as the average annualized volatility. All skewness are negative (but one) albeit very close to zero which indicates that the probability distribution is symmetric or very close to it. Finally, excess kurtosis are all positive, denoting tails heavier than Gaussian. The open and international markets have the highest excess kurtosis—the top 5 indices are in the US, South Korea, Netherlands and Canada.

**Table 2:** Descriptive statistics.

Index	Country	Median	IQR_0.75_	Kurtosis	Skewness
S&P	US	0.0006	0.0104	1.8598	-0.0034
NASDAQ	US	0.0009	0.0137	1.9299	-0.0036
TSX	Canada	0.0007	0.0098	1.6225	-0.0042
Merval	Argentina	0.0013	0.0221	1.4193	0.0008
Bovespa	Brazil	0.0009	0.0200	0.5942	-0.0029
IPC	Mexico	0.0006	0.0126	1.1945	-0.0020
AEX	Netherlands	0.0006	0.0126	1.6462	-0.0036
ATX	Austria	0.0007	0.0141	1.3296	-0.0053
FTSE	UK	0.0005	0.0113	1.4377	-0.0028
DAX	Germany	0.0008	0.0143	1.3958	-0.0055
CAC	France	0.0004	0.0138	1.3717	-0.0038
SMI	Spain	0.0006	0.0112	1.2319	-0.0035
MIB	Italy	0.0007	0.0151	1.2464	-0.0038
HgSg	Hong Kong	0.0006	0.0142	1.2157	-0.0025
Nikkei	Japan	0.0006	0.0155	0.9529	-0.0030
StrTim	Singapore	0.0002	0.0106	1.5006	-0.0036
SSEC	China	0.0008	0.0144	1.5891	-0.0050
BSE	India	0.0009	0.0140	1.4791	-0.0045
KLSE	Malaysia	0.0004	0.0077	1.4068	-0.0012
KOSPI	South Korea	0.0006	0.0134	1.8703	-0.0036
AllOrd	Australia	0.0007	0.0096	1.1289	-0.0034

All the metrics are quantile-based. *IQR*_75_ is the interquartile range, kurtosis is in excess and computed as *IQR*^0.975^/*IQR*^0.75^ − 2.91, and skewness is computed as (*Q*^0.975^ − *Q*^0.50^) − (*Q*^0.50^ − *Q*^0.025^).

We first show full-sample results, followed by rolling window estimations to study the dynamic behaviour. The window has a size of 3 years and it is rolled every year, i.e. we start with January 2000—December 2002, followed by January 2001—December 2003, and so forth. There are between 780 and 800 observations per window (except the last one that ends in July 2020). The choice of 3 years is motivated by having a relatively small sample size so we can compare the performance with the much larger full sample, but not too small that would not allow us to draw meaningful comparisons with competing measures.

Indeed, we compare TailCoR with the upper and lower exceedance correlations of [25] (denoted *θ*^+^(> *ξ*) and *θ*^−^(< *ξ*), respectively), and the parametric and non-parametric tail dependence coefficients (the former with a *t*-copula and the latter as in [11]). The non-parametric tail dependence coefficient is also called the co-exceedance probability; see equation (10) in [11]. We denote them *τ*_*p*_ and *τ*_*np*_, respectively. Results are for *ξ* = 0.975 (*ξ* = 0.025 for downside correlation). Since *τ*_*np*_ depends on the Hill statistic, alternative methods for choosing *ξ* are available. See [26] for a survey.

**Fig 8:**
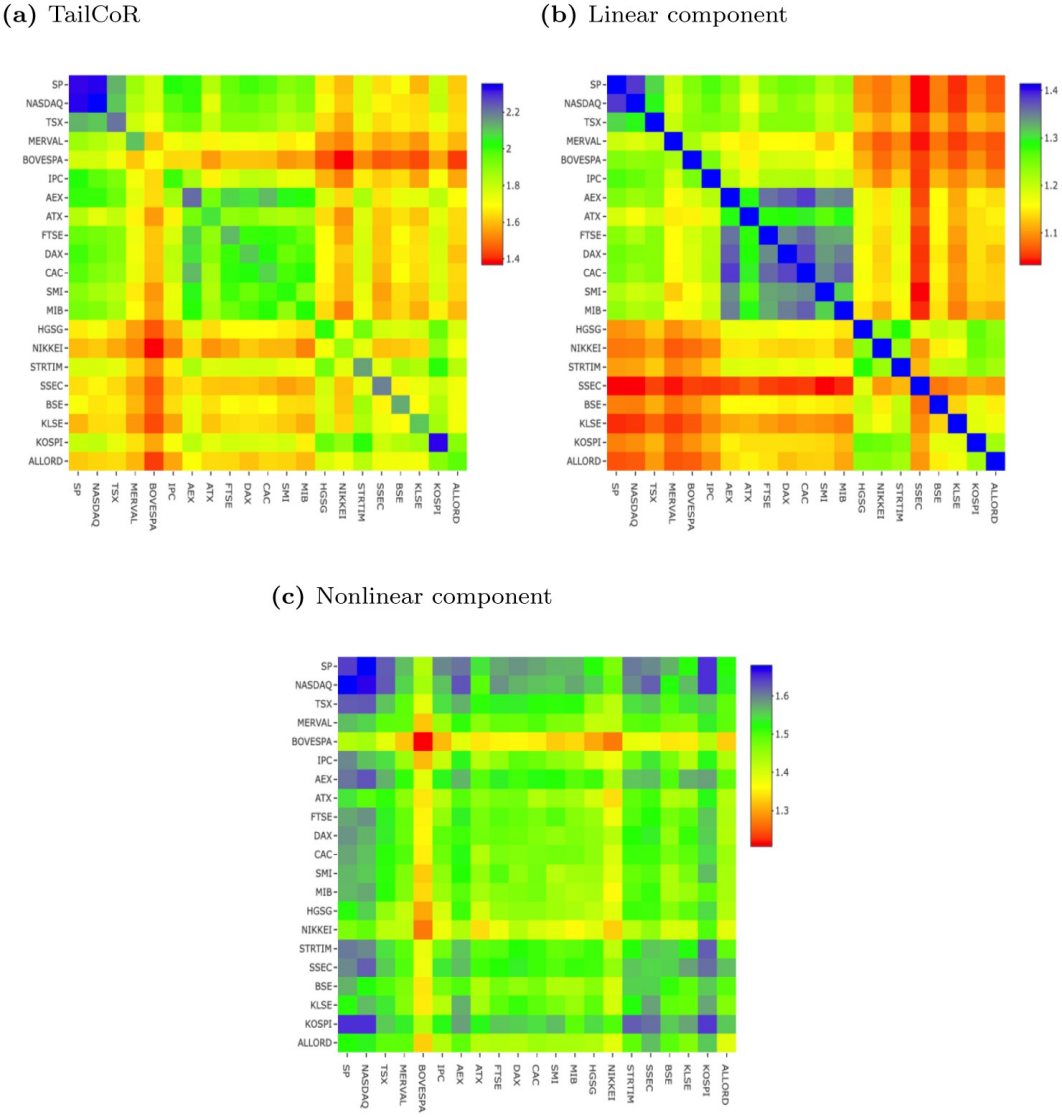
Full sample results—TailCoR and components. (a) TailCoR, (b) Linear component, (c) Nonlinear component.

**Fig 9:**
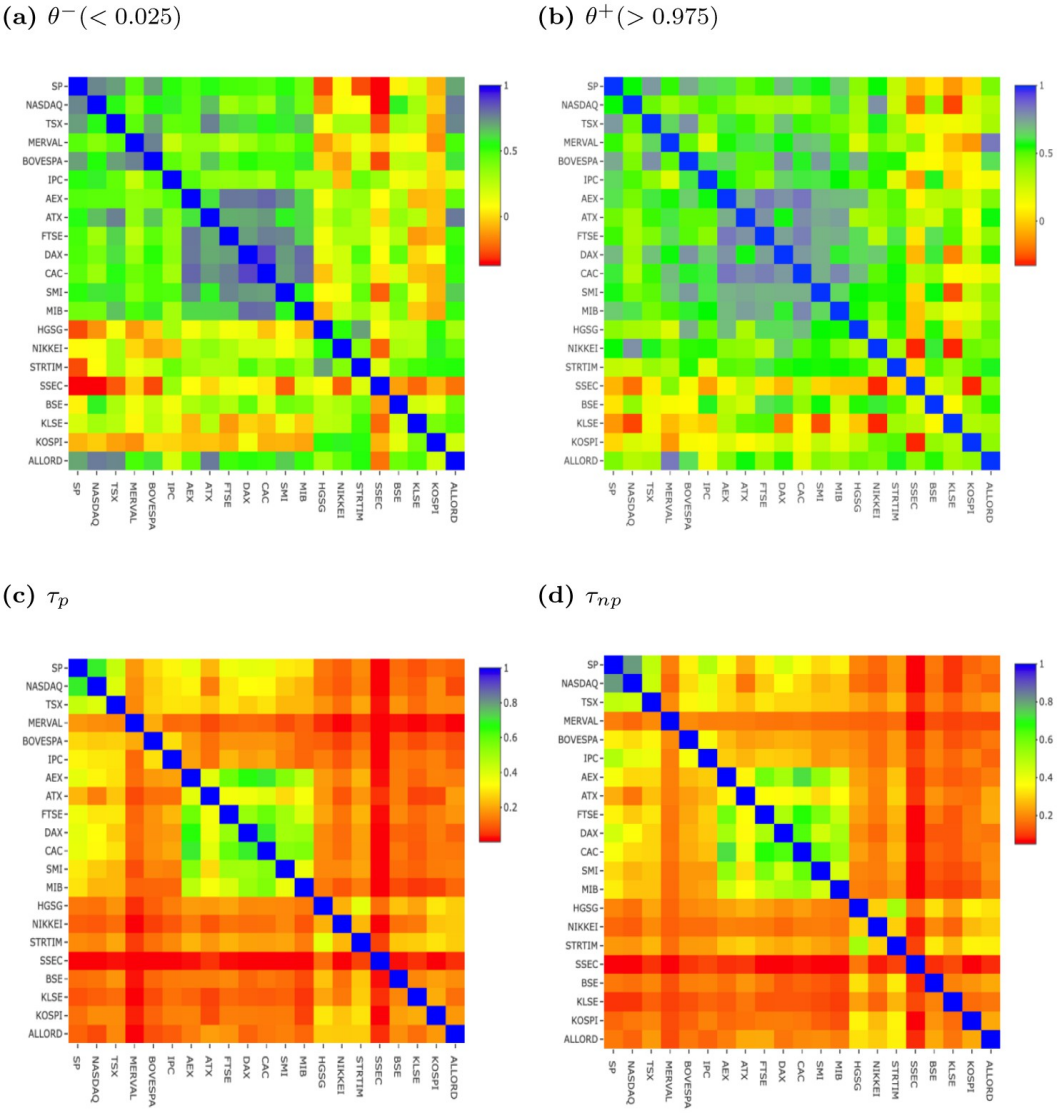
Full sample results—Comparing measures. (a) *θ*^−^ (< 0.025), (b) *θ*^+^ (> 0.975), (c) *τ*_*p*_, (d) *τ*_*np*_.

Full sample results for TailCoR and its components are summarized in Figs 8 and 9 in the form of heat maps. Detailed and tabulated full sample results are available in a separate S2 Appendix. The market indexes are ordered as in Table 2: America on the top left, Europe in the middle, and East Asia and Oceania in the bottom right. Note that the colour grading depends on the scale, but in all cases the closer to dark blue the stronger the dependence, and the closer to dark red the weaker the dependence.

Regarding TailCoR in heap map (a), all estimates are well above one—the minimum is 1.37—and with small standard deviations (between 0.04 and 0.13) reflecting the fact that markets are dependent and with heavy tails. As expected, for every market the diagonal elements are the highest. These elements measure TailCoR of one market with itself, which can be interpreted as a measure of tail risk. The markets with the highest tail risk are S&P and Nasdaq, as well as AEX and Kospi. The two latter are the Dutch and South Korean markets, that is two small and developed open economies. We address this point in more detail further below.

The average TailCoR of an index with the rest of world varies from 1.57 for Bovespa to 1.87 for AEX. More interestingly, the heat map shows a clear geographical clustering. The most dependent markets are in North America (S&P and NASDAQ), in dark blue and with TailCoRs above 2.1. By contrast, the remaining American markets have TailCoR of about 1.7. Interestingly enough, Latin American markets are less related between them than with North American markets. This is a sign of the leadership of US markets, that drives the overall performance of other American markets. It is also a sign of the lack of financial integration in Latin America. [27] finds that Latin America’s financial markets are less regionally integrated than in other regions of the world. Two reasons are behind this fact. First, many Latin American countries imposed restrictions in the movement of capital after the crises in the 80s and 90s. Second, as a consequence of the great financial crisis, many global financial institutions retrenched from the region.

The next block is Europe that is mostly in the green & dark green range (TailCoR is in the range 1.8–2.2). After North America, this is the second block with most dependent markets. Note that all markets but the UK belong to the European Union. In contrast with Latin America, European markets are relatively integrated as the free movement of capital is one of the four fundamental freedoms of the European Union, though more integration might be forthcoming if the Capital Markets Union regulation of the European Commission is approved. Among these markets, the most related is AEX—Netherlands is a very open economy and a European cross road—and the less is ATX—Austria is the furthest geographically.

The third block is Asia and Oceania with relatively low TailCoRs. The most dependent market is KOSPI—South Korea is another small and open economy, and very export oriented. Hence, there might be a relation between openness and TailCoR. A measure of openness in financial markets is the investment freedom, or the capacity of investment capital of individuals and firms to flow in a given market. The Heritage Foundation and the Wall Street Journal publish every year the investment freedom index that ranges from 0 (no freedom) to 100 (maximum freedom)—see www.heritage.org. Its correlation with TailCoR is 66%. This is a high correlation and confirms that markets with high openness entails high correlation of extreme events.

The heat map for the linear component reveals that the source of the geographical cluster is linear. All components are above one, with a minimum of 1.03 and a maximum of 1.39 (diagonal elements are excluded since, by definition, equal 1.41), and all standard deviations are very small. Within the blocks, and on average, the European block shows the highest linear dependence (1.22), followed by North America (1.18), Latin America (1.15), and Asia and Oceania (1.13). The correlation between the index of investment freedom and the linear component is 80%, i.e. the high correlation of 66% found earlier is driven by the linear component.

The heat map for the nonlinear component shows a different pattern. All nonlinear components are above one, standard deviations are small, and values do not show the same clear-cut cluster as for the linear components. That said, North American markets have the highest nonlinear relations, not only between them but also with the rest of the world. Some Asian indexes also show high nonlinear components, even higher than for European indexes, like KOSPI, SSEC, and STRTIM.

In order to give an empirical explanation to the nonlinear component, we need to consider two facts. First, the main diagonal measures the thickness of the tails for each market. In fact, the correlation of the main diagonal with the kurtosis in Table 2 is 99.8%. Second, the correlation between the index of investment freedom and the nonlinear component is just 12.5%. Therefore, while investment freedom explains the relation of markets in normal times, the nonlinear component captures the relation between markets during periods of global extreme events when contagion spreads, causing extreme co-movements. This finding is corroborated in the rolling window exercise.

Turning to the competing measures, *τ*_*p*_ and *τ*_*np*_ show the same geographical clustering as TailCoR, though the contrast between and within regional blocks is sharper, in particular for *τ*_*p*_ (as for TailCoR, detailed and tabulated full sample results are available in a separate S2 Appendix). This clustering is also visible in the heat map for *θ*^−^(< 0.025), though more blurred, and less visible for *θ*^+^(> 0.975). Table 3 displays the matrix of sample correlations between TailCoR^0.975^, *θ*^+^(> 0.975), *θ*^−^(< 0.025), *τ*_*p*_, and *τ*_*np*_. The sample correlations are computed by vectorizing the upper triangle of the heat maps, and calculating the Pearson correlations between them. The correlations of TailCoR with the other measures vary between 0.39 and 0.89. TailCoR highly correlates with *τ*_*p*_ and *τ*_*np*_, mildly correlates with *θ*^−^(< 0.025), and weakly correlates with *θ*^+^(> 0.975) (as a matter of consistency, one can see that *τ*_*np*_ and *τ*_*p*_ strongly or mildly correlate with all other measures). Overall, we conclude that for large samples TailCoR provides empirical results that are in line with tail dependence coefficients.

**Table 3:** Empirical correlations between all measures.

	TailCoR^0.975^	*θ*^−^(< 0.025)	*θ*^+^(> 0.975)	*τ*_*p*_	*τ*_*np*_
TailCoR^0.975^	1.00	0.51	0.39	0.84	0.80
*θ*^−^(< 0.025)	0.51	1.00	0.59	0.69	0.70
*θ*^+^(> 0.975)	0.39	0.59	1.00	0.57	0.61
*τ*_*p*_	0.84	0.69	0.57	1.00	0.96
*τ*_*np*_	0.80	0.70	0.61	0.96	1.00

Empirical correlations between TailCoR^0.975^, *θ*^+^(> 0.975), *θ*^−^(< 0.025), *τ*_*p*_, and *τ*_*np*_. The correlations are computed by vectorizing the upper triangle of the matrices of estimates, and calculating the Pearson correlations between them.

**Fig 10:**
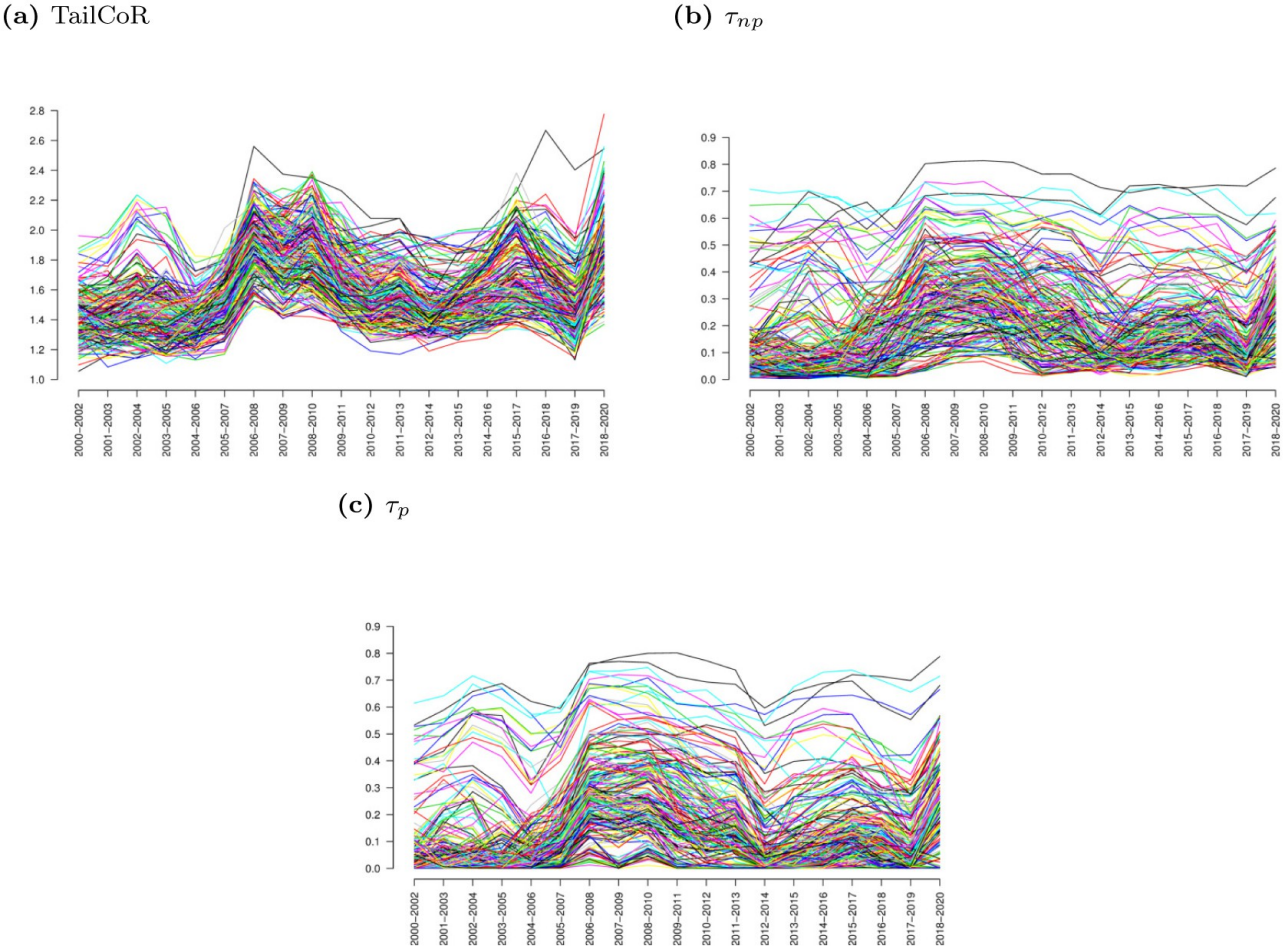
TailCoR and tail dependence coefficients. Panel (a) shows the evolution of TailCoR for all pairs of market indexes, while panels (b) and (c) show the non-parametric and parametric tail dependence coefficients.

Next, we move to the rolling window exercise. Panels (a), (b) and (c) of Fig 10 display the dynamic evolution of TailCoR, *τ*_*p*_, and *τ*_*np*_ respectively and for all pairs of market indexes. The exceedance correlations could not be computed due to lack of extreme observations. All TailCoRs are always larger than one, and they show a pattern in line with the financial and economic events that happened during the sample period—as explained in detail below. The tail dependence coefficients also show a recognisable pattern, though there is a larger variability across coefficients, both cross-sectionally and across time. For instance, we do not observe increased clustering in times of crisis.

**Fig 11:**
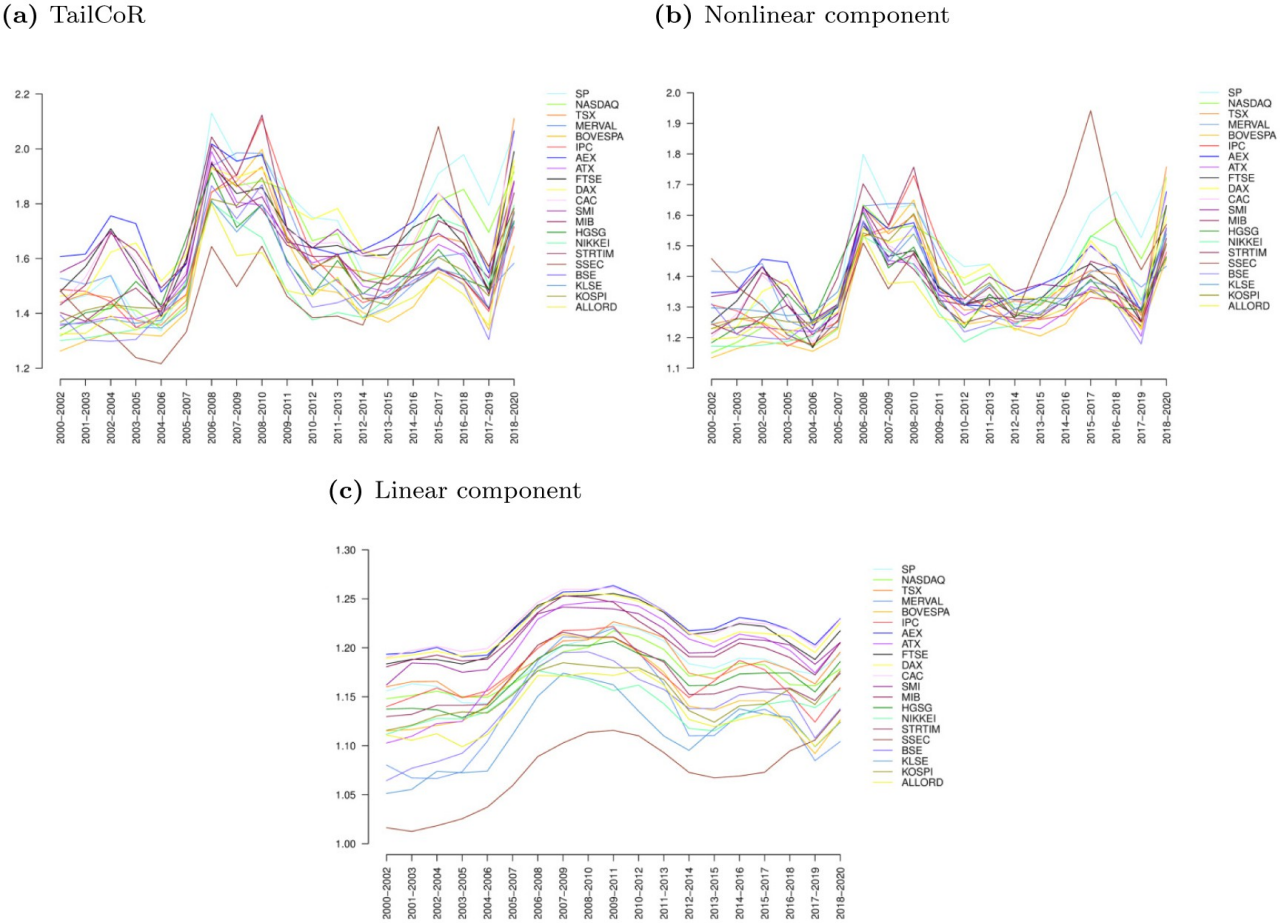
TailCoR and its components. Panel (a) shows the evolution of TailCoR for all market indexes. Each line is the cross-sectional average of one market index with respect to the others. Panels (b) and (c) show the evolution of the nonlinear and linear components respectively.

Fig 11 focuses on TailCoR (panel a) and its nonlinear (panel b) and linear components (panel c). For the sake of visibility and interpretation, each line is the cross-sectional average of one index with respect to the others. For instance, the value of TailCoR for S&P in 2000–2002 is the average TailCoR of S&P with respect to all the other indexes on that period.

Also here we observe that all TailCoRs are larger than 1 for all windows. The ups and downs show a pattern that is clearly identified with financial, economic and political events. In general TailCoR increases in crises periods, when markets become more dependent. Related with this, the variability of TailCoRs across the globe decreases in crises. During 2000–2004, the range of TailCoRs is 1.2–1.7. In 2005, all TailCoRs decrease, concentrating around 1.4. This a period characterised by tails nearly Gaussian. Since then, the heaviness and the tail co-movements increase steadily. March 2007 has been identified as the beginning of the financial crisis [28]. TailCoR decreases between 2007 and 2008, but picks up again in 2008, when further troubles in the financial industry were made public. TailCoR then lowers for the next three years, though there is a small peak around 2011–2012 due to the European sovereign debt crisis. These are years of bonanza in financial markets and the economy overall. In 2016, there were a number of corrections worldwide that are reflected in the increase of TailCoR. The last increase, to levels comparable to the 2008 Great Financial Crisis, is due to COVID-19.

Panels (b) and (c) reveal that the pattern of TailCoR is driven by the nonlinear component. The linear component evolves smoothly while the nonlinear component is more volatile and reacts faster to the events that drive financial markets. COVID-19 is a case in point. Though the linear component increases in the last window, the nonlinear component increases significantly more. Also, note that the linear component has an upward trend, an indication of the increasing integration of global financial markets. This trend is not observed in the nonlinear component. Instead, the nonlinear component is much more concentrated, which relates to the above-mentioned fact that in globally stressful periods, contagion spreads and we observe extreme co-movements.

## 5 Conclusions

We have introduced TailCoR, a metric for tail correlations that is a function of linear and nonlinear components, the latter characterised by the heaviness of the tails. TailCoR is exact for any probability level, it does not depend on any specific distributional assumption, and no optimisations are needed. Monte Carlo simulations reveal its goodness in finite samples.

The empirical application shows the reliability of TailCoR and its many potential uses for policy, namely as simple and quick tool for monitoring the propagation of large events in nonlinear systems. In our application to global equity market indexes, TailCoR brings light on the nature of the dependencies between markets in periods of stress. While there are clear geographical clusters when the dependence is linear, the nonlinearities caused by the most extreme dependencies are homogeneous, indicating that the extreme events evenly spread throughout the markets.

Several technical extensions are possible. One is conditional TailCoR, which would be based on the conditional IQR of the projection (${\text{IQR}}_{t}^{(j\;k)\;\xi }={\text{Q}}_{t}^{(j\;k)\;1-\xi }-{\text{Q}}_{t}^{(j\;k)\;\xi }$), where the time varying quantiles are regressed on the financial and economic determinants of tail correlations. Under ellipticity the dynamic linear correlation *ρ*_*ij*,*t*_ can be estimated with a robustified version of the DCC model ([29]).

A second extension is assuming a meta-elliptical copula ([30, 31]), which allows for different tail indexes for the marginal distributions and the copula. In this case, the first step should be done with a monotonically increasing transformation that standardises not only for the location and scale but also for the marginal heaviness of the tails.

## Supporting information

S1 Appendix Contains an appendix with all proofs. (PDF)

S2 Appendix Separate appendix with additional lengthy tables for consultation. (PDF)

## References

[1] Kiriliouk A, Rootzen H, Wadsworth J, Segers J. The meta-elliptical distributions with given marginals. Journal of Multivariate Analysis. 2019;82:1–16.

[2] Rootzen H, Segers J, Wadsworth J. Multivariate generalized Pareto distributions: parametrizations, representations, and properties. Journal of Multivariate Analysis. 2018;165:117–131. doi: 10.1016/j.jmva.2017.12.003

[3] Joe H. Multivariate Models and Dependence Concepts. New York: Chapman & Hall/CRC; 1997.

[4] Embrechts P, de Haan L, Huang X. Modeling multivariate extremes. In: Embrechts P, editor. Extremes and Integrated Risk Management. London: Risk books; 2000. p. 59–67.

[5] Beirlant J, Goegebeur Y, Segers J, Teugels J. Statistics of Extremes. Theory and Applications. New Jersey: John Wiley & Sons; 2004.

[6] McNeil AJ, Frey R, Paul E. Quantitative Risk Management: Concepts, Techniques, and Tools. Princeton University Press; 2005.

[7] Chollete L, de la Pena V, Lu CC. International diversification: A copula approach. Journal of Banking and Finance. 2011;35:403–417. doi: 10.1016/j.jbankfin.2010.08.020

[8] Hartmann P, Straetmans S, de Vries C. Asset Market Linkages in Crisis Periods. Review of Economics and Statistics. 2004;86:313–326. doi: 10.1162/003465304323023831

[9] Longin F, Solnik B. Extreme Correlation of International Equity Market. Journal of Finance. 2001;56:649–676. doi: 10.1111/0022-1082.00340

[10] Cizeau P, Potters M, Bouchaud JP. Correlation Structure of Extreme Stock Returns. Quantitative Finance. 2001;1:217–222. doi: 10.1080/713665669

[11] Straetmans S, Verschoor W, Wolf C. Extreme US stock market fluctuations in the wake of 9/11. Journal of Applied Econometrics. 2008;23:17–42. doi: 10.1002/jae.973

[12] Ledford A, Tawn J. Statistics for near independence in multivariate extreme values. Biometrika. 1996;83:169–187. doi: 10.1093/biomet/83.1.169

[13] Hartmann P, Straetmans S, de Vries C. Banking System Stability: A Cross-Atlantic Perspective. In: Carey M, Stulz RM, editors. Risk of Financial Institutions. Chicago: The University of Chicago Press; 2005. p. 133–193.

[14] Poon SH, Rockinger M, Tawn J. Extreme Value Dependence in Financial Markets: Diagnosis, Models, and Financial Implications. Review of Financial Studies. 2004;17:581–610. doi: 10.1093/rfs/hhg058

[15] Babić S, Ley C, Veredas D. Comparison and Classification of Flexible Distributions for Multivariate Skew and Heavy-Tailed Data. Symmetry. 2019;11:1216. doi: 10.3390/sym11101216

[16] Forbes F, Wraith D. Location and scale mixtures of Gaussians with flexible tail behaviour: Properties, inference and application to multivariate clustering. Statistics and Computing. 2015;90:61–73. doi: 10.1016/j.csda.2015.04.008

[17] Fang HB, Fanga KT, Kotz S. The meta-elliptical distributions with given marginals. Journal of Multivariate Analysis. 2002;82:1–16. doi: 10.1006/jmva.2001.2017

[18] Lee TH, Long X. Copula-based Multivariate GARCH Models with Uncorrelated Dependent Errors. Journal of Econometrics. 2019;150:207–218. doi: 10.1016/j.jeconom.2008.12.008

[19] Sherman S. A theorem on convex sets with applications. The Annals of Mathematical Statistics. 1955;26:763–767. doi: 10.1214/aoms/1177728435

[20] Berkes I, Hörmann S, Schauer J. Asymptotic Results for the Empirical Process of Stationary Sequences. Stochastic Processes and their Applications. 2009;119:1298–1324. doi: 10.1016/j.spa.2008.06.010

[21] Hashorva E. Tail asymptotic results for elliptical distributions. Insurance: Mathematics and Economics. 2008;43:158–164.

[22] Hashorva E. On the residual dependence index of elliptical distributions. Statistics & Probability Letters. 2010;80:1070–1078. doi: 10.1016/j.spl.2010.03.001

[23] McCulloch J. Simple consistent estimators of stable distribution parameters. Commun Statist Simula. 1986;15:1109–1136. doi: 10.1080/03610918608812563

[24] Lindskog F, McNeil A, Schmock U. Kendall’s tau for Elliptical Distributions. In: Bol G, Gholamreza Nakhaeizadeh TR Svetlozar T Rachev, Vollmer KR, editors. Credit Risk—Measurement, Evaluation and Management. Physica–Verlag HD; 2003.

[25] Longin FM, Solnik B. Extreme Correlations of International Equity Markets. Journal of Finance. 2001;24:1097–1130.

[26] Dominicy Y, Ilmonen P, Veredas D. Multivariate Hill Estimators. International Statistical Review. 2017;85:108–142. doi: 10.1111/insr.12120

[27] IMF. Financial Integration in Latin America. Staff Report. 2016.

[28] Acharya VV, Richardson M. Restoring Financial Stability. New Jersey: John Willey & Sons; 2009.

[29] Boudt K, Danielsson J, Laurent S. Robust Forecasting of Dynamic Conditional Correlation GARCH Models. International Journal of Forecasting. 2013;29:244–257. doi: 10.1016/j.ijforecast.2012.06.003

[30] Klüppelberg C, Kuhn G, Peng L. Semi-parametric models for the multivariate tail dependence function – the asymptotically dependent case. Scandinavian Journal of Statistics. 2008;35:701–718. doi: 10.1111/j.1467-9469.2008.00602.x

[31] Krajina A. A method of moments estimator of tail dependence in meta-elliptical models. Journal of Statistical Planning and Inference. 2012;142:1811–1823. doi: 10.1016/j.jspi.2012.01.020

