# Supplementary material for: TailCoR: A new and simple metric for tail correlations that disentangles the linear and nonlinear dependencies that cause extreme co-movements
Source: PLoS One. 2023 Jan 3;18(1):e0278599. doi: 10.1371/journal.pone.0278599 (PMC9810202; doi:10.1371/journal.pone.0278599)
Supplement: S1 Appendix — (PDF) [file pone.0278599.s001.pdf]

# Appendix to TailCoR: A new and simple metric for tail correlations that disentangles the linear and nonlinear dependencies that cause extreme co-movements

Sladana Babić[1], Christophe Ley*[2], Lorenzo Ricci[3], David Veredas[4],

**1** LeasePlan, Amsterdam, The Netherlands
**2** Department of Mathematics, University of Luxembourg, Esch-sur-Alzette, Luxembourg
**3** European Stability Mechanism, Luxembourg, Luxembourg
**4** Centre for Sustainable Finance and Department of Economics, Vlerick Business School and Ghent University, Brussels, Belgium

* christophe.ley@uni.lu

## Proof of Theorem 1

We first consider the case when $\rho_{j\,k} > 0$ and hence $Z_t^{(j\,k)} = \frac{1}{\sqrt{2}}(Y_{j\,t} + Y_{k\,t})$. The variance of $Y_{j\,t}$ is

$$\sigma^2_{Y_j} = \frac{\sigma^2_{X_j}}{(\mathrm{IQR}^\tau_j)^2},$$

and likewise for $Y_{k\,t}$. The variance of $Z_t^{(j\,k)}$ is

$$\sigma^2_{(j\,k)} = \frac{1}{2}\left(\frac{\sigma^2_{X_j}}{(\mathrm{IQR}^\tau_j)^2} + \frac{\sigma^2_{X_k}}{(\mathrm{IQR}^\tau_k)^2} + 2\sigma_{Y_jY_k}\right),$$

where $\sigma_{Y_jY_k}$ is the covariance between $Y_{j\,t}$ and $Y_{k\,t}$. Since $\mathrm{IQR}^\tau_j = k(\tau,\alpha)\sigma_{X_j}$ and $\mathrm{IQR}^\tau_k = k(\tau,\alpha)\sigma_{X_k}$, we find

$$\sigma^2_{(j\,k)} = \frac{1}{2}\left(\frac{\sigma^2_{X_j}}{k(\tau,\alpha)^2\sigma^2_{X_j}} + \frac{\sigma^2_{X_k}}{k(\tau,\alpha)^2\sigma^2_{X_k}} + 2\frac{\sigma_{X_j\,X_k}}{k(\tau,\alpha)^2\sigma_{X_j}\sigma_{X_k}}\right),$$

which, in a more compact form, equals

$$\sigma^2_{(j\,k)} = \frac{1}{k(\tau,\alpha)^2}\left(1 + \rho_{j\,k}\right).$$

By the affine invariance of the elliptical family, $\mathrm{IQR}^{(j\,k)\,\xi} = k(\xi,\alpha)\sigma_{(j\,k)}$. Substituting in $\sigma^2_{(j\,k)}$

$$\mathrm{IQR}^{(j\,k)\,\xi} = \frac{k(\xi,\alpha)}{k(\tau,\alpha)}\sqrt{1+\rho_{j\,k}} = s(\xi,\tau,\alpha)\sqrt{1+\rho_{j\,k}}.$$

In the Gaussian case $k(\tau,\alpha) = k(\tau)$ and $k(\xi,\alpha) = k(\xi)$. We normalize $\mathrm{IQR}^{(j\,k)\xi}$ by $\frac{k(\tau)}{k(\xi)} = s_g(\xi,\tau)$ yielding

$$\mathrm{TailCoR}^{(j\,k)\,\xi} = s_g(\xi,\tau)s(\xi,\tau,\alpha)\sqrt{1+\rho_{j\,k}}.$$

The same proof follows for $\rho_{j\,k} < 0$ and $Z_t^{(j\,k)} = \frac{1}{\sqrt{2}}(Y_{j\,t} - Y_{k\,t})$, except that $\sqrt{1+\rho_{j\,k}}$ is replaced by $\sqrt{1-\rho_{j\,k}}$. This change is unsubstantial since both expressions are equal ($\rho_{j\,k}$ is positive in $\sqrt{1+\rho_{j\,k}}$ and negative in $\sqrt{1-\rho_{j\,k}}$). Hence

$$\mathrm{TailCoR}^{(j\,k)\,\xi} = s_g(\xi,\tau)s(\xi,\tau,\alpha)\sqrt{1+|\rho_{j\,k}|}.$$

Q.E.D.

## Proof of Theorem 2

Let $\mathrm{Q}_{j_T}^{0.50} := \mathrm{Q}_j^{0.50} + T^{-\frac{1}{2}}\eta_T^1$ for some bounded sequence $\eta_T^1$ and $\mathrm{Q}_{k_T}^{0.50} := \mathrm{Q}_k^{0.50} + T^{-\frac{1}{2}}\eta_T^2$ for some bounded sequence $\eta_T^2$. Similarly, let $\mathrm{IQR}_{j_T}^\tau := \mathrm{IQR}_j^\tau + T^{-\frac{1}{2}}s_T^1$ and $\mathrm{IQR}_{k_T}^\tau = \mathrm{IQR}_k^\tau + T^{-\frac{1}{2}}s_T^2$ for some bounded sequences $s_T^1$ and $s_T^2$.

First, we show boundedness of the estimated IQR.

**Lemma 1** For a given data set $X_1, \ldots, X_n$ and some values $a_1, \ldots, a_n$ that belong to the interval $[-\epsilon, \epsilon]$, we can define $Y_i := X_i + a_i$. Denote the interquantile range of the data set $X$ and $Y$ by $\hat{\mathrm{IQR}}(X_1, \ldots, X_n)$ and $\hat{\mathrm{IQR}}(Y_1, \ldots, Y_n)$, respectively. Then the following holds:

$$|\hat{\mathrm{IQR}}(X_1, \ldots, X_n) - \hat{\mathrm{IQR}}(Y_1, \ldots, Y_n)| \leq 10\epsilon.$$

**Proof** Let $\sigma$ be a permutation that orders our data $X$ in ascending order. That is, $X_{\sigma(1)} \leq X_{\sigma(2)} \leq \cdots \leq X_{\sigma(n)}$. Similarly, $\pi$ is a permutation such that $Y_{\pi(1)} \leq Y_{\pi(2)} \leq \cdots \leq Y_{\pi(n)}$. Then, for fixed values $i$ and $j$ we have that $\hat{\mathrm{IQR}}(X_1, \ldots, X_n) = X_{\sigma(j)} - X_{\sigma(i)}$ and $\hat{\mathrm{IQR}}(Y_1, \ldots, Y_n) = Y_{\pi(j)} - Y_{\pi(i)}$. First we will show that $|X_{\sigma(i)} - Y_{\pi(i)}| \leq 5\epsilon$ for all $i$. Let $k$ be such that $\pi(k) = \sigma(i)$ and let $m$ be such that $\pi(i) = \sigma(m)$. We have two cases:

- $k, m < i$ or $k, m > i$. That means that data points $X_{\sigma(i)}$ and $X_{\sigma(m)}$ changed their ordering after we added the noise. This implies that $|X_{\sigma(i)} - X_{\sigma(m)}| \leq 2\epsilon$. Therefore, $|X_{\sigma(i)} - Y_{\pi(i)}| = |X_{\sigma(i)} - X_{\sigma(m)} - a_{\sigma(m)}| \leq |X_{\sigma(i)} - X_{\sigma(m)}| + |a_{\sigma(m)}| \leq 3\epsilon$.

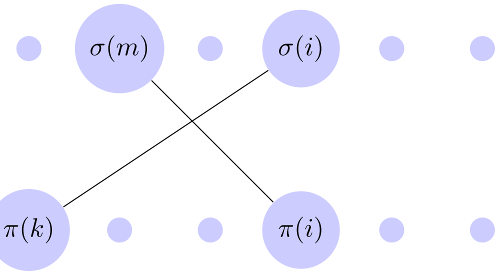


- $k < i < m$ or $m < i < k$ . In this case we have that the ordering is preserved which means $X_{\sigma(m)} \geq X_{\sigma(i)} \wedge Y_{\pi(i)} \geq Y_{\pi(k)}$ or $X_{\sigma(m)} \leq X_{\sigma(i)} \wedge Y_{\pi(i)} \leq Y_{\pi(k)}$. Assume that we have the first case.

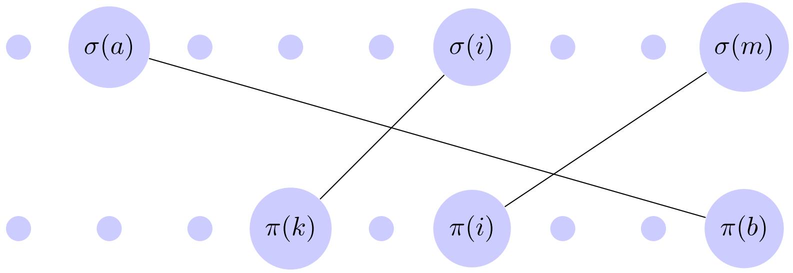


The ordering of the elements $X_{\sigma(i)}$ and $X_{\sigma(m)}$ is preserved but these elements shifted after adding the noise. In our example, $X_{\sigma(m)}$ was not in the first $i$ elements originally, but after adding the noise, it is there. This implies that there is an element, let us say $X_{\sigma(a)}$, that originally was in the first $i$ elements but after adding the noise it is not there anymore. That is, $a < i < m$. Therefore, there is $b$ such that $k < i < b$. We can conclude that $X_{\sigma(a)}$ has changed its ordering with respect to both $X_{\sigma(i)}$ and $X_{\sigma(m)}$. This implies that $|X_{\sigma(i)} - X_{\sigma(a)}| \leq 2\epsilon$ and $|X_{\sigma(m)} - X_{\sigma(a)}| \leq 2\epsilon$. Hence,

$$|X_{\sigma(i)} - Y_{\pi(i)}| = |X_{\sigma(i)} - X_{\sigma(m)} - a_{\sigma(m)}| \leq |X_{\sigma(i)} - X_{\sigma(m)}| + |a_{\sigma(m)}| \leq$$
$$|X_{\sigma(i)} - X_{\sigma(a)} + X_{\sigma(a)} - X_{\sigma(m)}| + \epsilon \leq |X_{\sigma(i)} - X_{\sigma(a)}| + |X_{\sigma(a)} - X_{\sigma(m)}| + \epsilon \leq 2\epsilon + 2\epsilon + \epsilon = 5\epsilon$$

Finally,

$$|\hat{\text{IQR}}(X_1,\ldots,X_n) - \hat{\text{IQR}}(Y_1,\ldots,Y_n)| = |X_{\sigma(j)} - X_{\sigma(i)} - (Y_{\pi(j)} - Y_{\pi(i)})| \leq$$
$$|X_{\sigma(j)} - Y_{\pi(j)}| + |X_{\sigma(i)} - Y_{\pi(i)}| \leq 10\epsilon.$$

Q.E.D.

We now turn to the proof of Theorem 2. We need to prove that

$$\text{Tail}\hat{\text{Co}}\text{R}_T^{(jk)\xi}(\hat{\text{Q}}_j^{0.50}, \hat{\text{Q}}_k^{0.50}, \hat{\text{IQR}}_j^\tau, \hat{\text{IQR}}_k^\tau) - \text{TailCoR}^{(jk)\xi}$$

is $o_p(1)$. First, we add and subtract $\text{Tail}\hat{\text{Co}}\text{R}_T^{(jk)\xi}$ as a function of the population quantiles:

$$\begin{aligned}\text{Tail}\hat{\text{Co}}\text{R}_T^{(jk)\xi}(\hat{\text{Q}}_j^{0.50}, \hat{\text{Q}}_k^{0.50}, \hat{\text{IQR}}_j^\tau, \hat{\text{IQR}}_k^\tau) - \text{Tail}\hat{\text{Co}}\text{R}_T^{(jk)\xi}(\text{Q}_j^{0.50}, \text{Q}_k^{0.50}, \text{IQR}_j^\tau, \text{IQR}_k^\tau) \\ + \text{Tail}\hat{\text{Co}}\text{R}_T^{(jk)\xi}(\text{Q}_j^{0.50}, \text{Q}_k^{0.50}, \text{IQR}_j^\tau, \text{IQR}_k^\tau) - \text{TailCoR}^{(jk)\xi}.\end{aligned} \tag{1}$$

We will first prove that the first part of (1) is $o_p(1)$, that is, as $T \to \infty$

$$\text{Tail}\hat{\text{Co}}\text{R}_T^{(jk)\xi}(\hat{\text{Q}}_j^{0.50}, \hat{\text{Q}}_k^{0.50}, \hat{\text{IQR}}_j^\tau, \hat{\text{IQR}}_k^\tau) - \text{Tail}\hat{\text{Co}}\text{R}_T^{(jk)\xi}(\text{Q}_j^{0.50}, \text{Q}_k^{0.50}, \text{IQR}_j^\tau, \text{IQR}_k^\tau) = o_p(1).$$

The definition of TailCoR implies that we have to prove that

$$\hat{\text{IQR}}_T^{(jk)\xi}(\hat{\text{Q}}_j^{0.50}, \hat{\text{Q}}_k^{0.50}, \hat{\text{IQR}}_j^\tau, \hat{\text{IQR}}_k^\tau) - \hat{\text{IQR}}_T^{(jk)\xi}(\text{Q}_j^{0.50}, \text{Q}_k^{0.50}, \text{IQR}_j^\tau, \text{IQR}_k^\tau) = o_p(1)$$

as $T \to \infty$. In view of Lemma 4.4 of [1], this reduces to proving that

$$\hat{\text{IQR}}_T^{(jk)\xi}(\text{Q}_{j_T}^{0.50}, \text{Q}_{k_T}^{0.50}, \text{IQR}_{j_T}^\tau, \text{IQR}_{k_T}^\tau) - \hat{\text{IQR}}_T^{(jk)\xi}(\text{Q}_j^{0.50}, \text{Q}_k^{0.50}, \text{IQR}_j^\tau, \text{IQR}_k^\tau) = o_p(1)$$

as $T \to \infty$, for the sequences defined at the very beginning of the proof. We add and subtract $\hat{\text{IQR}}_T^{(jk)\xi}$ as a function of the population quantiles:

$$\begin{aligned}&\hat{\text{IQR}}_T^{(jk)\xi}(\text{Q}_{j_T}^{0.50}, \text{Q}_{k_T}^{0.50}, \text{IQR}_{j_T}^\tau, \text{IQR}_{k_T}^\tau) - \hat{\text{IQR}}_T^{(jk)\xi}(\text{Q}_j^{0.50}, \text{Q}_k^{0.50}, \text{IQR}_{j_T}^\tau, \text{IQR}_{k_T}^\tau) \\ &+ \hat{\text{IQR}}_T^{(jk)\xi}(\text{Q}_j^{0.50}, \text{Q}_k^{0.50}, \text{IQR}_{j_T}^\tau, \text{IQR}_{k_T}^\tau) - \hat{\text{IQR}}_T^{(jk)\xi}(\text{Q}_j^{0.50}, \text{Q}_k^{0.50}, \text{IQR}_j^\tau, \text{IQR}_k^\tau).\end{aligned} \tag{2}$$

We now show that $\hat{\text{IQR}}_T^{(jk)\xi}(\text{Q}_{j_T}^{0.50}, \text{Q}_{k_T}^{0.50}, \text{IQR}_{j_T}^\tau, \text{IQR}_{k_T}^\tau) - \hat{\text{IQR}}_T^{(jk)\xi}(\text{Q}_j^{0.50}, \text{Q}_k^{0.50}, \text{IQR}_{j_T}^\tau, \text{IQR}_{k_T}^\tau) = 0$. Indeed, $Y_{jt}^{(1)} = \frac{X_{jt} - \text{Q}_{j_T}^{0.50}}{\text{IQR}_{j_T}^\tau} = Y_{jt}^{(2)} - \frac{T^{-\frac{1}{2}}\eta_T^1}{\text{IQR}_{j_T}^\tau}$ where $Y_{jt}^{(2)} = \frac{X_{jt} - \text{Q}_j^{0.50}}{\text{IQR}_{j_T}^\tau}$ and
$Y_{kt}^{(1)} = \frac{X_{kt} - \text{Q}_{k_T}^{0.50}}{\text{IQR}_{k_T}^\tau} = Y_{kt}^{(2)} - \frac{T^{-\frac{1}{2}}\eta_T^2}{\text{IQR}_{k_T}^\tau}$ where $Y_{kt}^{(2)} = \frac{X_{kt} - \text{Q}_k^{0.50}}{\text{IQR}_{k_T}^\tau}$. Therefore,

$$\begin{aligned}Z_t^{(1)(jk)} &= Y_{jt}^{(1)} \cos\phi + Y_{kt}^{(1)} \sin\phi \\ &= \left(Y_{jt}^{(2)} - \frac{T^{-\frac{1}{2}}\eta_T^1}{\text{IQR}_{j_T}^\tau}\right) \cos\phi + \left(Y_{kt}^{(2)} - \frac{T^{-\frac{1}{2}}\eta_T^2}{\text{IQR}_{k_T}^\tau}\right) \sin\phi \\ &= Y_{jt}^{(2)} \cos\phi + Y_{kt}^{(2)} \sin\phi - \frac{T^{-\frac{1}{2}}\eta_T^1}{\text{IQR}_{j_T}^\tau} \cos\phi - \frac{T^{-\frac{1}{2}}\eta_T^2}{\text{IQR}_{k_T}^\tau} \sin\phi \\ &= Z_t^{(2)(jk)} - \frac{T^{-\frac{1}{2}}\eta_T^1}{\text{IQR}_{j_T}^\tau} \cos\phi - \frac{T^{-\frac{1}{2}}\eta_T^2}{\text{IQR}_{k_T}^\tau} \sin\phi.\end{aligned}$$

This implies that the IQR of $Z_t^{(1)(jk)}$ equals the IQR of $Z_t^{(2)(jk)}$. The next step is to prove that

$$\hat{\mathrm{IQR}}_T^{(jk)\xi}(\mathrm{Q}_j^{0.50}, \mathrm{Q}_k^{0.50}, \mathrm{IQR}_{j_T}^{\tau}, \mathrm{IQR}_{k_T}^{\tau}) - \hat{\mathrm{IQR}}_T^{(jk)\xi}(\mathrm{Q}_j^{0.50}, \mathrm{Q}_k^{0.50}, \mathrm{IQR}_j^{\tau}, \mathrm{IQR}_k^{\tau}) = o_p(1)$$

as $T \to \infty$. The notation $\hat{\mathrm{IQR}}_T^{(jk)\xi}(\mathrm{Q}_j^{0.50}, \mathrm{Q}_k^{0.50}, \mathrm{IQR}_{j_T}^{\tau}, \mathrm{IQR}_{k_T}^{\tau})$ means that we have to estimate the IQR of the following sample

$$\frac{X_{j1} - \mathrm{Q}_j^{0.50}}{\mathrm{IQR}_{j_T}^{\tau}} \cos\phi + \frac{X_{k1} - \mathrm{Q}_k^{0.50}}{\mathrm{IQR}_{k_T}^{\tau}} \sin\phi, \dots, \frac{X_{jT} - \mathrm{Q}_j^{0.50}}{\mathrm{IQR}_{j_T}^{\tau}} \cos\phi + \frac{X_{kT} - \mathrm{Q}_k^{0.50}}{\mathrm{IQR}_{k_T}^{\tau}} \sin\phi.$$

Let $T$ be large enough such that $\mathrm{IQR}_{j_T}^{\tau} = \mathrm{IQR}_j^{\tau} + \frac{s_T^1}{\sqrt{T}} > \frac{\mathrm{IQR}_j^{\tau}}{2}$ and $\mathrm{IQR}_{k_T}^{\tau} = \mathrm{IQR}_k^{\tau} + \frac{s_T^2}{\sqrt{T}} > \frac{\mathrm{IQR}_k^{\tau}}{2}$. For such $T$ we have that:

$$\left|\frac{1}{\mathrm{IQR}_j^{\tau} + \frac{s_T^1}{\sqrt{T}}} - \frac{1}{\mathrm{IQR}_j^{\tau}}\right| = \frac{1}{\sqrt{T}}\left|\frac{s_T^1}{\mathrm{IQR}_j^{\tau}(\mathrm{IQR}_j^{\tau} + \frac{s_T^1}{\sqrt{T}})}\right| < \frac{1}{\sqrt{T}}\frac{2|s_T^1|}{(\mathrm{IQR}_j^{\tau})^2} \le \frac{1}{\sqrt{T}}\frac{2C_1}{(\mathrm{IQR}_j^{\tau})^2}.$$

Similarly, $\left|\frac{1}{\mathrm{IQR}_k^{\tau} + \frac{s_T^2}{\sqrt{T}}} - \frac{1}{\mathrm{IQR}_k^{\tau}}\right| \le \frac{1}{\sqrt{T}}\frac{2C_2}{(\mathrm{IQR}_k^{\tau})^2}$. So, for any $t < T$ and $T$ large enough we have that

$$\begin{aligned}
&\left|\frac{X_{jt} - \mathrm{Q}_j^{0.50}}{\mathrm{IQR}_{j_T}^{\tau}} \cos\phi + \frac{X_{kt} - \mathrm{Q}_k^{0.50}}{\mathrm{IQR}_{k_T}^{\tau}} \sin\phi - \frac{X_{jt} - \mathrm{Q}_j^{0.50}}{\mathrm{IQR}_j^{\tau}} \cos\phi - \frac{X_{kt} - \mathrm{Q}_k^{0.50}}{\mathrm{IQR}_k^{\tau}} \sin\phi\right| \\
\le& \frac{1}{\sqrt{T}}\frac{2C_1}{(\mathrm{IQR}_j^{\tau})^2}\left|X_{jt} - \mathrm{Q}_j^{0.50}\right||\cos\phi| + \frac{1}{\sqrt{T}}\frac{2C_2}{(\mathrm{IQR}_k^{\tau})^2}\left|X_{kt} - \mathrm{Q}_k^{0.50}\right||\sin\phi| \\
\le& \frac{1}{\sqrt{T}}\frac{2C_1}{(\mathrm{IQR}_j^{\tau})^2}\max_t\left|X_{jt}\right||\cos\phi| + \frac{1}{\sqrt{T}}\frac{2C_2}{(\mathrm{IQR}_k^{\tau})^2}\max_t\left|X_{kt}\right||\sin\phi| \\
+& \frac{1}{\sqrt{T}}\frac{2C_1}{(\mathrm{IQR}_j^{\tau})^2}\left|\mathrm{Q}_j^{0.50}\right||\cos\phi| + \frac{1}{\sqrt{T}}\frac{2C_2}{(\mathrm{IQR}_k^{\tau})^2}\left|\mathrm{Q}_k^{0.50}\right||\sin\phi|.
\end{aligned}$$

Using Lemma 1 and the upper bound obtained above, we can conclude that

$$\begin{aligned}
&\left|\hat{\mathrm{IQR}}_T^{(jk)\xi}(\mathrm{Q}_j^{0.50}, \mathrm{Q}_k^{0.50}, \mathrm{IQR}_{j_T}^{\tau}, \mathrm{IQR}_{k_T}^{\tau}) - \hat{\mathrm{IQR}}_T^{(jk)\xi}(\mathrm{Q}_j^{0.50}, \mathrm{Q}_k^{0.50}, \mathrm{IQR}_j^{\tau}, \mathrm{IQR}_k^{\tau})\right| \\
\le& 10\left(\frac{1}{\sqrt{T}}\frac{2C_1}{(\mathrm{IQR}_j^{\tau})^2}\max_t\left|X_{jt}\right||\cos\phi| + \frac{1}{\sqrt{T}}\frac{2C_2}{(\mathrm{IQR}_k^{\tau})^2}\max_t\left|X_{kt}\right||\sin\phi| \right.\\
&\left. + \frac{1}{\sqrt{T}}\frac{2C_1}{(\mathrm{IQR}_j^{\tau})^2}\left|\mathrm{Q}_j^{0.50}\right||\cos\phi| + \frac{1}{\sqrt{T}}\frac{2C_2}{(\mathrm{IQR}_k^{\tau})^2}\left|\mathrm{Q}_k^{0.50}\right||\sin\phi|\right)
\end{aligned}$$

Since

$$\begin{aligned}
&\frac{1}{\sqrt{T}}\frac{2C_1}{(\mathrm{IQR}_j^{\tau})^2}\max_t\left|X_{jt}\right||\cos\phi| + \frac{1}{\sqrt{T}}\frac{2C_2}{(\mathrm{IQR}_k^{\tau})^2}\max_t\left|X_{kt}\right||\sin\phi| + \\
&+ \frac{1}{\sqrt{T}}\frac{2C_1}{(\mathrm{IQR}_j^{\tau})^2}\left|\mathrm{Q}_j^{0.50}\right||\cos\phi| + \frac{1}{\sqrt{T}}\frac{2C_2}{(\mathrm{IQR}_k^{\tau})^2}\left|\mathrm{Q}_k^{0.50}\right||\sin\phi| = o_p(1)
\end{aligned}$$

as $T \to \infty$, it holds that

$$\hat{\mathrm{IQR}}_T^{(jk)\xi}(\mathrm{Q}_j^{0.50}, \mathrm{Q}_k^{0.50}, \mathrm{IQR}_{j_T}^{\tau}, \mathrm{IQR}_{k_T}^{\tau}) - \hat{\mathrm{IQR}}_T^{(jk)\xi}(\mathrm{Q}_j^{0.50}, \mathrm{Q}_k^{0.50}, \mathrm{IQR}_j^{\tau}, \mathrm{IQR}_k^{\tau}) = o_p(1)$$

as $T \to \infty$. Last, we need to prove that the second part of (1) is $o_p(1)$, in other words, that

$$\mathrm{Tail}\hat{\mathrm{Co}}\mathrm{R}_T^{(jk)\xi}(\mathrm{Q}_j^{0.50}, \mathrm{Q}_k^{0.50}, \mathrm{IQR}_j^{\tau}, \mathrm{IQR}_k^{\tau}) - \mathrm{TailCoR}^{(jk)\xi} = o_p(1)$$

as $T \to \infty$. This follows from the asymptotic properties of sample quantiles under $S$–mixing ( [2]). Q.E.D.

### Proof of Theorem 3

By **E1**, $\text{Tail}\hat{\text{Co}}\text{R}_T^{(j\,k)\,\xi} = 2s_g(\xi,\tau)\hat{\text{Q}}_T^{(j\,k)\,\xi}$. The term $2s_g(\xi,\tau)$ is a deterministic scale shift and the only source of randomness is $\hat{\text{Q}}_T^{(j\,k)\,\xi}$. Hence

$$\begin{aligned} E(\text{Tail}\hat{\text{Co}}\text{R}_T^{(j\,k)\,\xi}) &= 2s_g(\xi,\tau)E(\hat{\text{Q}}_T^{(j\,k)\,\xi}) \text{ and} \\ Var(\text{Tail}\hat{\text{Co}}\text{R}_T^{(j\,k)\,\xi}) &= 4s_g(\xi,\tau)^2 Var(\hat{\text{Q}}_T^{(j\,k)\,\xi}). \end{aligned}$$

By the asymptotic properties of sample quantiles under $S$–mixing ( [2]) and the delta method the proof is completed. Q.E.D.

## References


1. Kreiss JP. On adaptive estimation in stationary ARMA processes. The Annals of Statistics. 1987;15(1):112–133.

2. Dominicy Y, Hörmann S, Ogata H, Veredas D. On Sample Marginal Quantiles for Stationary Processes. Statistics and Probability Letters. 2013;83:28–36.
