# Supplementary material for: TailCoR: A new and simple metric for tail correlations that disentangles the linear and nonlinear dependencies that cause extreme co-movements
Source: PLoS One. 2023 Jan 3;18(1):e0278599. doi: 10.1371/journal.pone.0278599 (PMC9810202; doi:10.1371/journal.pone.0278599)
Supplement: S2 Appendix — (PDF) [file pone.0278599.s002.pdf]

Appendix T to TailCoR: A new and simple metric for tail correlations that disentangles the linear and nonlinear dependencies that cause extreme co-movements

Sladana Babić[1], Christophe Ley*[2], Lorenzo Ricci[3], David Veredas[4],

1 LeasePlan, Amsterdam, The Netherlands
2 Department of Mathematics, University of Luxembourg, Esch-sur-Alzette, Luxembourg
3 European Stability Mechanism, Luxembourg, Luxembourg
4 Centre for Sustainable Finance and Department of Economics, Vlerick Business School and Ghent University, Brussels, Belgium

* christophe.ley@uni.lu

Table 1. Tabulation of $s_g(\xi, \tau)$

	ξ													
τ	0.700	0.725	0.750	0.775	0.800	0.825	0.850	0.875	0.900	0.925	0.950	0.975	0.990	0.995
0.600	0.483	0.424	0.375	0.335	0.301	0.271	0.244	0.220	0.198	0.176	0.154	0.129	0.109	0.098
0.625	0.607	0.533	0.472	0.422	0.379	0.341	0.307	0.277	0.249	0.221	0.194	0.163	0.137	0.124
0.650	0.735	0.644	0.571	0.510	0.458	0.412	0.372	0.335	0.301	0.268	0.234	0.196	0.166	0.150
0.675	0.865	0.759	0.673	0.601	0.539	0.486	0.438	0.394	0.354	0.315	0.276	0.231	0.195	0.176
0.700	–	0.877	0.778	0.694	0.623	0.561	0.506	0.456	0.409	0.364	0.319	0.267	0.226	0.204
0.725	–	–	0.886	0.791	0.711	0.640	0.577	0.520	0.466	0.415	0.363	0.305	0.257	0.232
0.750	–	–	–	0.893	0.801	0.722	0.651	0.586	0.526	0.468	0.410	0.344	0.290	0.262
0.775	–	–	–	–	0.898	0.808	0.729	0.657	0.589	0.525	0.459	0.385	0.325	0.293
0.800	–	–	–	–	–	0.900	0.812	0.731	0.657	0.585	0.512	0.429	0.362	0.327
0.825	–	–	–	–	–	–	0.902	0.812	0.729	0.649	0.568	0.477	0.402	0.363
0.850	–	–	–	–	–	–	–	0.901	0.809	0.720	0.630	0.529	0.445	0.402
0.875	–	–	–	–	–	–	–	–	0.898	0.799	0.699	0.587	0.494	0.447
0.900	–	–	–	–	–	–	–	–	–	0.890	0.779	0.654	0.551	0.497

Interpolation can be used for values of ξ and τ that are not in the table. Alternatively, $s_g(\xi, \tau)$ can be computed as $\frac{\Phi(\tau)^{-1}}{\Phi(\xi)^{-1}}$ where $\Phi(\cdot)$ is the cumulative distribution function of a standardized Gaussian distribution.

Table 2. Matrix of $\text{TailCoR}^{0.975}$

	SP	NASDAQ	TSX	MERVAL	BOVESPA	IPC	AEX	ATX	FTSE	DAX	CAC	SMI	MIB	HGSG	NIKKEI	STRTIM	SSEC	BSE	KLSE	KOSPI	ALLORD	Average
SP	2.33	2.33	2.12	1.87	1.78	2.03	2.00	1.83	1.95	1.99	1.97	1.91	1.93	1.67	1.59	1.77	1.65	1.71	1.59	1.80	1.62	1.86
	(13.69)	(12.89)	(13.53)	(8.77)	(7.87)	(10.59)	(10.43)	(10.22)	(10.11)	(10.17)	(10.49)	(9.93)	(8.98)	(8.28)	(7.68)	(9.78)	(7.92)	(9.21)	(8.38)	(8.91)	(9.70)	
NASDAQ		2.36	2.10	1.83	1.78	1.97	2.00	1.74	1.93	1.95	1.92	1.86	1.91	1.71	1.61	1.77	1.68	1.66	1.64	1.81	1.63	1.84
		(12.85)	(12.15)	(7.56)	(6.65)	(10.08)	(9.50)	(10.18)	(8.55)	(9.07)	(9.32)	(8.80)	(6.84)	(7.65)	(6.96)	(9.17)	(7.49)	(7.55)	(7.98)	(7.67)	(6.53)	
TSX			2.21	1.79	1.72	1.92	1.94	1.80	1.90	1.90	1.88	1.82	1.85	1.65	1.57	1.74	1.64	1.67	1.65	1.74	1.64	1.80
			(15.55)	(7.31)	(8.76)	(10.22)	(10.89)	(9.91)	(10.36)	(9.66)	(9.86)	(9.84)	(8.59)	(9.48)	(7.52)	(9.61)	(8.97)	(10.34)	(8.92)	(9.22)	(9.01)	
MERVAL				2.11	1.61	1.72	1.75	1.68	1.72	1.72	1.73	1.66	1.70	1.55	1.52	1.63	1.56	1.59	1.54	1.65	1.59	1.67
				(10.84)	(5.87)	(6.93)	(7.19)	(8.49)	(6.23)	(6.52)	(5.94)	(7.03)	(6.17)	(5.35)	(6.39)	(8.00)	(6.11)	(6.64)	(5.69)	(6.24)	(7.17)	
BOVESPA					1.71	1.64	1.64	1.55	1.61	1.61	1.60	1.54	1.56	1.45	1.37	1.53	1.46	1.47	1.44	1.57	1.42	1.57
					(6.26)	(7.37)	(6.26)	(7.35)	(6.44)	(6.40)	(6.84)	(6.28)	(4.57)	(5.87)	(4.65)	(6.05)	(7.19)	(8.89)	(5.70)	(7.32)	(6.52)	
IPC						2.00	1.85	1.68	1.76	1.81	1.80	1.73	1.74	1.58	1.51	1.67	1.62	1.65	1.56	1.71	1.56	1.73
						(10.03)	(8.67)	(8.36)	(10.28)	(7.97)	(8.85)	(9.18)	(6.47)	(6.91)	(6.44)	(8.43)	(7.21)	(10.15)	(7.26)	(7.97)	(7.04)	
AEX							2.22	1.93	2.08	2.06	2.12	2.04	2.02	1.77	1.64	1.85	1.66	1.74	1.75	1.82	1.70	1.87
							(11.65)	(8.50)	(9.93)	(9.46)	(11.01)	(10.86)	(7.90)	(6.29)	(6.72)	(8.26)	(6.67)	(7.23)	(9.02)	(9.09)	(8.70)	
ATX								2.06	1.89	1.90	1.86	1.84	1.86	1.64	1.54	1.76	1.61	1.65	1.61	1.74	1.64	1.74
								(12.88)	(9.75)	(9.27)	(9.43)	(8.52)	(8.89)	(7.85)	(7.97)	(10.79)	(6.11)	(10.15)	(8.59)	(7.90)	(9.28)	
FTSE									2.12	2.00	2.01	1.99	1.95	1.70	1.57	1.77	1.61	1.71	1.65	1.78	1.64	1.81
									(8.07)	(7.66)	(9.22)	(9.38)	(7.42)	(6.66)	(5.86)	(7.97)	(6.83)	(7.96)	(6.89)	(8.13)	(7.40)	
DAX										2.10	2.03	1.95	1.99	1.69	1.62	1.78	1.61	1.67	1.65	1.77	1.61	1.82
										(9.71)	(9.23)	(9.05)	(7.58)	(6.60)	(6.48)	(8.04)	(5.42)	(6.13)	(6.86)	(7.48)	(7.23)	
CAC											2.08	1.99	2.02	1.70	1.59	1.78	1.59	1.70	1.64	1.76	1.64	1.82
											(10.31)	(9.62)	(7.53)	(6.25)	(6.03)	(8.38)	(6.22)	(7.58)	(6.87)	(7.60)	(6.68)	
SMI												2.02	1.89	1.67	1.59	1.74	1.56	1.65	1.62	1.78	1.63	1.77
												(9.82)	(7.22)	(5.46)	(6.85)	(8.58)	(6.31)	(6.66)	(6.97)	(7.92)	(7.91)	
MIB													2.02	1.64	1.51	1.72	1.58	1.64	1.58	1.67	1.59	1.77
													(7.60)	(5.46)	(5.49)	(8.43)	(6.31)	(5.90)	(6.38)	(7.13)	(6.95)	
HGSG														2.01	1.72	1.94	1.78	1.76	1.78	1.95	1.77	1.71
														(9.41)	(7.31)	(12.40)	(8.06)	(9.48)	(7.78)	(8.32)	(9.28)	
NIKKEI															1.88	1.74	1.60	1.61	1.63	1.88	1.72	1.61
															(7.63)	(8.29)	(7.38)	(8.37)	(6.21)	(7.27)	(7.38)	
STRTIM																2.15	1.74	1.86	1.84	2.01	1.82	1.77
																(13.08)	(7.35)	(10.85)	(8.39)	(9.72)	(9.31)	
SSEC																	2.19	1.68	1.73	1.79	1.72	1.64
																	(9.10)	(6.96)	(6.63)	(7.15)	(7.51)	
BSE																		2.14	1.69	1.83	1.72	1.68
																		(10.52)	(7.99)	(9.10)	(8.87)	
KLSE																			2.10	1.86	1.72	1.66
																			(8.35)	(7.25)	(7.20)	
KOSPI																				2.33	1.90	1.79
																				(11.10)	(7.76)	
ALLORD																					1.97	1.66
																					(9.45)	

Table 3. Matrix of linear contributions $\sqrt{1+|\rho|}$

	SP	NASDAQ	TSX	MERVAL	BOVESPA	IPC	AEX	ATX	FTSE	DAX	CAC	SMI	MIB	HGSG	NIKKEI	STRTIM	SSEC	BSE	KLSE	KOSPI	ALLORD	Average
SP	1.41 (0.00)	1.39 (0.18)	1.31 (0.50)	1.20 (1.01)	1.24 (0.76)	1.27 (0.62)	1.25 (0.59)	1.19 (0.88)	1.24 (0.61)	1.25 (0.67)	1.25 (0.64)	1.22 (0.71)	1.23 (0.63)	1.10 (0.74)	1.08 (0.71)	1.10 (0.61)	1.03 (0.80)	1.09 (0.73)	1.05 (0.76)	1.09 (0.65)	1.06 (0.84)	1.18
NASDAQ		1.41 (0.00)	1.29 (0.51)	1.18 (1.03)	1.23 (0.86)	1.26 (0.60)	1.23 (0.58)	1.17 (0.93)	1.22 (0.68)	1.24 (0.58)	1.23 (0.62)	1.20 (0.78)	1.21 (0.59)	1.10 (0.66)	1.09 (0.69)	1.11 (0.63)	1.04 (0.81)	1.09 (0.73)	1.05 (0.84)	1.10 (0.64)	1.07 (0.69)	1.17
TSX			1.41 (0.00)	1.20 (1.08)	1.24 (0.83)	1.24 (0.75)	1.24 (0.60)	1.19 (0.87)	1.24 (0.60)	1.24 (0.62)	1.24 (0.62)	1.20 (0.75)	1.22 (0.69)	1.13 (0.72)	1.10 (0.74)	1.13 (0.71)	1.06 (0.79)	1.11 (0.83)	1.07 (0.84)	1.12 (0.70)	1.10 (0.77)	1.18
MERVAL				1.41 (0.00)	1.22 (0.96)	1.19 (0.93)	1.16 (1.07)	1.15 (1.04)	1.16 (1.07)	1.16 (1.05)	1.16 (1.03)	1.13 (0.98)	1.15 (1.04)	1.10 (0.83)	1.07 (0.89)	1.09 (0.86)	1.04 (0.94)	1.08 (0.88)	1.05 (0.86)	1.08 (0.78)	1.06 (0.82)	1.13
BOVESPA					1.41 (0.00)	1.25 (0.77)	1.18 (0.76)	1.15 (0.91)	1.19 (0.75)	1.19 (0.84)	1.19 (0.81)	1.16 (0.84)	1.17 (0.80)	1.11 (0.75)	1.08 (0.80)	1.10 (0.81)	1.06 (0.78)	1.10 (1.03)	1.07 (0.89)	1.10 (0.76)	1.07 (0.80)	1.15
IPC						1.41 (0.00)	1.21 (0.67)	1.18 (0.82)	1.21 (0.71)	1.21 (0.73)	1.22 (0.70)	1.19 (0.86)	1.20 (0.79)	1.13 (0.75)	1.09 (0.64)	1.13 (0.77)	1.05 (0.83)	1.12 (0.96)	1.09 (0.76)	1.12 (0.72)	1.09 (0.72)	1.17
AEX							1.41 (0.00)	1.29 (0.79)	1.36 (0.34)	1.37 (0.25)	1.39 (0.19)	1.34 (0.42)	1.35 (0.37)	1.17 (0.68)	1.14 (0.60)	1.18 (0.69)	1.06 (0.81)	1.16 (0.93)	1.11 (0.88)	1.15 (0.62)	1.14 (0.76)	1.22
ATX								1.41 (0.00)	1.28 (0.78)	1.29 (0.87)	1.30 (0.78)	1.26 (0.72)	1.29 (0.77)	1.17 (0.77)	1.15 (0.78)	1.18 (0.80)	1.07 (0.87)	1.15 (1.04)	1.12 (0.95)	1.14 (0.82)	1.15 (0.85)	1.19
FTSE									1.41 (0.00)	1.34 (0.38)	1.36 (0.33)	1.33 (0.42)	1.33 (0.49)	1.17 (0.62)	1.14 (0.63)	1.18 (0.70)	1.06 (0.86)	1.16 (0.90)	1.10 (0.87)	1.14 (0.66)	1.14 (0.79)	1.22
DAX										1.41 (0.00)	1.38 (0.19)	1.34 (0.45)	1.35 (0.34)	1.16 (0.65)	1.13 (0.70)	1.17 (0.67)	1.05 (0.82)	1.15 (0.92)	1.10 (0.93)	1.14 (0.67)	1.12 (0.82)	1.22
CAC											1.41 (0.00)	1.35 (0.48)	1.37 (0.28)	1.16 (0.62)	1.14 (0.73)	1.18 (0.69)	1.05 (0.76)	1.15 (0.91)	1.10 (0.88)	1.14 (0.64)	1.13 (0.80)	1.22
SMI												1.41 (0.00)	1.31 (0.59)	1.16 (0.69)	1.14 (0.65)	1.17 (0.73)	1.04 (0.94)	1.15 (0.84)	1.10 (0.87)	1.14 (0.68)	1.13 (0.76)	1.20
MIB													1.41 (0.00)	1.15 (0.67)	1.11 (0.71)	1.15 (0.65)	1.05 (0.82)	1.14 (0.91)	1.09 (0.94)	1.12 (0.68)	1.11 (0.85)	1.21
HGSG														1.41 (0.00)	1.24 (0.61)	1.28 (0.66)	1.18 (0.93)	1.20 (0.87)	1.19 (0.88)	1.26 (0.60)	1.24 (0.72)	1.17
NIKKEI															1.41 (0.00)	1.23 (0.56)	1.10 (0.85)	1.15 (0.79)	1.17 (0.86)	1.25 (0.67)	1.24 (0.69)	1.14
STRTIM																1.41 (0.00)	1.12 (1.08)	1.20 (0.96)	1.21 (0.87)	1.24 (0.65)	1.22 (0.72)	1.17
SSEC																	1.41 (0.00)	1.08 (0.93)	1.09 (0.93)	1.11 (1.07)	1.10 (0.85)	1.07
BSE																		1.41 (0.00)	1.14 (1.02)	1.18 (0.78)	1.15 (0.81)	1.14
KLSE																			1.41 (0.00)	1.18 (0.93)	1.17 (0.95)	1.11
KOSPI																				1.41 (0.00)	1.22 (0.73)	1.15
ALLORD																					1.41 (0.00)	1.14

Table 4. Matrix of nonlinear contributions $s_g(0.975, 0.75)s(0.975, 0.75, \alpha)$

	SP	NASDAQ	TSX	MERVAL	BOVESPA	IPC	AEX	ATX	FTSE	DAX	CAC	SMI	MIB	HGSG	NIKKEI	STRTIM	SSEC	BSE	KLSE	KOSPI	ALLORD	Average
SP	1.64 (9.68)	1.68 (9.27)	1.62 (10.11)	1.56 (7.04)	1.43 (6.04)	1.60 (8.07)	1.61 (8.35)	1.54 (8.37)	1.58 (8.03)	1.59 (7.88)	1.58 (8.23)	1.57 (7.88)	1.57 (7.13)	1.52 (7.31)	1.47 (7.04)	1.61 (8.70)	1.60 (7.44)	1.57 (7.99)	1.52 (7.83)	1.65 (8.01)	1.52 (8.54)	1.57
NASDAQ		1.67 (9.09)	1.63 (9.24)	1.55 (6.08)	1.44 (5.15)	1.56 (7.86)	1.63 (7.76)	1.50 (8.33)	1.59 (6.91)	1.57 (7.18)	1.56 (7.54)	1.56 (7.16)	1.58 (5.57)	1.55 (6.82)	1.49 (6.49)	1.59 (8.18)	1.62 (7.06)	1.52 (6.53)	1.56 (7.44)	1.65 (6.90)	1.53 (5.82)	1.57
TSX			1.56 (11.00)	1.49 (5.97)	1.39 (6.63)	1.54 (7.88)	1.57 (8.67)	1.51 (7.96)	1.53 (8.14)	1.53 (7.62)	1.52 (7.70)	1.51 (7.84)	1.52 (6.83)	1.46 (8.14)	1.42 (6.60)	1.54 (8.32)	1.55 (8.28)	1.50 (8.87)	1.54 (8.25)	1.56 (8.15)	1.49 (7.86)	1.52
MERVAL				1.49 (7.67)	1.32 (4.69)	1.45 (5.58)	1.51 (5.98)	1.46 (6.88)	1.49 (5.04)	1.48 (5.40)	1.50 (4.81)	1.47 (5.87)	1.47 (5.10)	1.42 (4.64)	1.42 (5.62)	1.49 (7.05)	1.50 (5.79)	1.47 (5.81)	1.47 (5.28)	1.53 (5.63)	1.49 (6.42)	1.48
BOVESPA					1.21 (4.43)	1.31 (5.56)	1.39 (5.13)	1.34 (5.88)	1.35 (5.15)	1.36 (5.15)	1.35 (5.46)	1.33 (4.96)	1.34 (3.73)	1.30 (4.98)	1.27 (4.08)	1.38 (5.25)	1.38 (6.38)	1.35 (7.47)	1.35 (5.08)	1.43 (6.50)	1.33 (5.65)	1.36
IPC						1.41 (7.09)	1.53 (6.91)	1.43 (6.71)	1.46 (8.14)	1.49 (6.21)	1.48 (7.00)	1.46 (7.28)	1.45 (5.12)	1.40 (5.76)	1.38 (5.59)	1.48 (7.10)	1.54 (6.54)	1.47 (8.43)	1.43 (6.40)	1.53 (6.95)	1.43 (6.10)	1.47
AEX							1.57 (8.24)	1.50 (6.41)	1.53 (7.19)	1.51 (6.89)	1.53 (7.87)	1.52 (7.90)	1.49 (5.76)	1.51 (5.32)	1.43 (5.81)	1.56 (6.90)	1.56 (6.01)	1.50 (5.92)	1.57 (7.87)	1.58 (7.79)	1.49 (7.41)	1.53
ATX								1.46 (9.11)	1.48 (7.36)	1.48 (7.04)	1.43 (6.99)	1.45 (6.57)	1.44 (6.63)	1.40 (6.26)	1.34 (6.50)	1.49 (8.70)	1.51 (5.43)	1.43 (8.08)	1.44 (7.15)	1.53 (6.67)	1.43 (7.54)	1.46
FTSE									1.50 (5.71)	1.49 (5.64)	1.47 (6.65)	1.50 (6.88)	1.46 (5.50)	1.45 (5.55)	1.38 (4.93)	1.50 (6.69)	1.52 (6.15)	1.48 (6.57)	1.49 (5.94)	1.56 (6.88)	1.43 (6.05)	1.49
DAX										1.48 (6.86)	1.47 (6.64)	1.46 (6.67)	1.47 (5.56)	1.46 (5.56)	1.43 (5.53)	1.52 (6.65)	1.53 (5.03)	1.46 (4.93)	1.51 (5.87)	1.55 (6.36)	1.43 (6.13)	1.49
CAC											1.47 (7.29)	1.48 (6.92)	1.48 (5.46)	1.46 (5.23)	1.40 (5.00)	1.51 (6.90)	1.51 (5.62)	1.48 (6.08)	1.49 (5.84)	1.54 (6.51)	1.45 (5.52)	1.48
SMI												1.43 (6.94)	1.44 (5.31)	1.45 (4.67)	1.39 (5.85)	1.49 (7.08)	1.50 (5.82)	1.43 (5.47)	1.47 (6.15)	1.56 (6.93)	1.44 (6.70)	1.47
MIB													1.43 (5.38)	1.44 (4.58)	1.36 (4.57)	1.49 (7.02)	1.51 (5.76)	1.43 (4.68)	1.45 (5.50)	1.49 (6.16)	1.44 (5.75)	1.46
HGSG														1.42 (6.65)	1.39 (5.71)	1.51 (9.34)	1.51 (6.36)	1.46 (7.43)	1.49 (6.26)	1.55 (6.52)	1.43 (7.25)	1.46
NIKKEI															1.33 (5.40)	1.42 (6.73)	1.46 (6.46)	1.40 (6.92)	1.40 (5.06)	1.50 (5.92)	1.39 (5.76)	1.41
STRTIM																1.52 (9.25)	1.56 (6.07)	1.55 (8.49)	1.52 (6.74)	1.62 (7.70)	1.49 (7.45)	1.52
SSEC																	1.55 (6.43)	1.55 (6.01)	1.58 (5.71)	1.61 (6.08)	1.56 (6.53)	1.53
BSE																		1.51 (7.44)	1.49 (6.49)	1.56 (7.37)	1.49 (7.21)	1.48
KLSE																			1.49 (5.90)	1.58 (5.79)	1.47 (5.77)	1.49
KOSPI																				1.65 (7.85)	1.56 (6.36)	1.56
ALLORD																					1.39 (6.68)	1.46

Table 5. Downside exceedance correlation: $\theta^{-}(< 0.025)$

	SP	NASDAQ	TSX	MERVAL	BOVESPA	IPC	AEX	ATX	FTSE	DAX	CAC	SMI	MIB	HGSG	NIKKEI	STRTIM	SSEC	BSE	KLSE	KOSPI	ALLORD	Average
SP	1.00	0.76	0.72	0.47	0.71	0.53	0.48	0.55	0.49	0.49	0.46	0.53	0.42	-0.27	0.07	-0.27	-0.37	0.05	0.19	-0.07	0.70	0.33
NASDAQ		1.00	0.56	0.37	0.54	0.58	0.46	0.66	0.32	0.40	0.33	0.59	0.41	-0.13	0.10	0.09	-0.36	0.57	0.31	-0.02	0.78	0.36
TSX			1.00	0.46	0.71	0.45	0.46	0.77	0.62	0.60	0.56	0.58	0.65	0.11	0.30	0.31	-0.25	0.29	0.26	-0.11	0.76	0.44
MERVAL				1.00	0.77	0.22	0.36	0.35	0.25	0.34	0.42	0.37	0.49	-0.12	0.00	0.13	-0.02	0.12	0.06	-0.14	0.49	0.27
BOVESPA					1.00	0.45	0.37	0.64	0.46	0.53	0.49	0.49	0.58	-0.01	-0.10	0.20	-0.28	0.24	0.26	0.01	0.59	0.38
IPC						1.00	0.47	0.36	0.34	0.36	0.31	0.48	0.31	0.30	-0.05	0.43	0.33	0.12	0.15	-0.01	0.18	0.32
AEX							1.00	0.60	0.79	0.79	0.82	0.75	0.62	0.11	0.31	0.33	0.09	0.29	-0.06	-0.04	0.44	0.42
ATX								1.00	0.69	0.70	0.73	0.57	0.62	0.21	0.36	0.28	-0.00	0.34	0.27	-0.04	0.78	0.47
FTSE									1.00	0.72	0.77	0.76	0.62	0.11	0.29	0.29	0.13	0.28	-0.12	-0.07	0.50	0.41
DAX										1.00	0.88	0.72	0.81	0.14	0.20	0.48	0.09	0.45	-0.01	0.11	0.52	0.47
CAC											1.00	0.71	0.80	-0.04	0.21	0.34	0.03	0.19	-0.05	-0.08	0.51	0.42
SMI												1.00	0.57	0.07	0.11	0.45	-0.24	0.42	0.13	-0.07	0.64	0.43
MIB													1.00	-0.06	0.21	0.33	0.17	0.39	-0.03	-0.10	0.51	0.41
HGSG														1.00	0.52	0.71	0.10	0.24	0.25	0.52	0.30	0.15
NIKKEI															1.00	0.38	-0.24	0.29	0.27	0.57	0.44	0.21
STRTIM																1.00	0.17	0.42	0.49	0.53	0.55	0.33
SSEC																	1.00	-0.11	-0.22	-0.07	-0.20	-0.06
BSE																		1.00	0.20	0.15	0.43	0.27
KLSE																			1.00	0.40	0.49	0.16
KOSPI																				1.00	0.20	0.08
ALLORD																					1.00	0.48

Table 6. Upside exceedance correlation: $\theta^{+}(> 0.975)$

	SP	NASDAQ	TSX	MERVAL	BOVESPA	IPC	AEX	ATX	FTSE	DAX	CAC	SMI	MIB	HGSG	NIKKEI	STRTIM	SSEC	BSE	KLSE	KOSPI	ALLORD	Average
SP	1.00	0.56	0.77	0.53	0.73	0.63	0.61	0.47	0.52	0.70	0.60	0.57	0.66	0.41	0.56	0.29	-0.06	0.05	-0.12	0.02	0.45	0.45
NASDAQ		1.00	0.47	0.37	0.44	0.51	0.45	0.36	0.35	0.38	0.33	0.45	0.61	0.36	0.78	0.26	-0.21	0.40	-0.27	0.23	0.32	0.36
TSX			1.00	0.64	0.78	0.41	0.60	0.52	0.44	0.68	0.52	0.50	0.45	0.35	0.58	0.39	0.07	0.17	0.15	0.19	0.35	0.45
MERVAL				1.00	0.49	0.22	0.54	0.60	0.64	0.56	0.69	0.67	0.51	0.22	0.47	0.33	0.43	0.13	-0.05	-0.12	0.83	0.44
BOVESPA					1.00	0.60	0.66	0.49	0.50	0.77	0.58	0.76	0.59	0.72	0.53	0.54	0.14	0.11	0.04	0.14	0.62	0.51
IPC						1.00	0.57	0.50	0.42	0.65	0.60	0.61	0.56	0.47	0.54	0.20	-0.10	0.58	-0.02	0.11	0.15	0.41
AEX							1.00	0.72	0.83	0.78	0.84	0.71	0.70	0.69	0.42	0.50	0.09	0.31	0.33	0.22	0.44	0.55
ATX								1.00	0.78	0.57	0.79	0.73	0.75	0.34	0.40	0.48	0.30	0.25	0.48	0.12	0.56	0.51
FTSE									1.00	0.75	0.82	0.71	0.72	0.63	0.31	0.61	0.05	0.62	0.50	0.18	0.41	0.54
DAX										1.00	0.77	0.72	0.59	0.63	0.66	0.53	-0.05	0.60	-0.17	0.29	0.53	0.55
CAC											1.00	0.72	0.80	0.69	0.50	0.56	0.33	0.40	0.13	0.14	0.21	0.55
SMI												1.00	0.64	0.56	0.54	0.54	0.02	0.56	-0.26	0.29	0.42	0.52
MIB													1.00	0.55	0.43	0.55	-0.03	0.28	0.16	0.19	0.37	0.50
HGSG														1.00	0.33	0.53	-0.03	0.54	-0.02	0.31	0.47	0.44
NIKKEI															1.00	0.36	-0.33	0.59	-0.33	0.47	0.44	0.41
STRTIM																1.00	0.02	0.51	0.37	0.50	0.58	0.43
SSEC																	1.00	0.22	0.33	-0.32	0.41	0.06
BSE																		1.00	0.12	0.39	0.53	0.37
KLSE																			1.00	0.33	0.35	0.10
KOSPI																				1.00	0.41	0.20
ALLORD																					1.00	0.44

Table 7. t-copula tail dependence: τ_p

	SP	NASDAQ	TSX	MERVAL	BOVESPA	IPC	AEX	ATX	FTSE	DAX	CAC	SMI	MIB	HGSG	NIKKEI	STRTIM	SSEC	BSE	KLSE	KOSPI	ALLORD	Average
SP	1.00	0.69	0.44	0.18	0.28	0.32	0.38	0.22	0.36	0.38	0.37	0.30	0.29	0.13	0.10	0.16	0.01	0.12	0.08	0.12	0.10	0.25
NASDAQ		1.00	0.40	0.17	0.25	0.30	0.32	0.14	0.30	0.33	0.32	0.26	0.22	0.12	0.09	0.15	0.00	0.13	0.10	0.16	0.07	0.23
TSX			1.00	0.16	0.26	0.29	0.29	0.24	0.30	0.26	0.29	0.23	0.23	0.17	0.12	0.20	0.03	0.17	0.12	0.16	0.16	0.23
MERVAL				1.00	0.21	0.11	0.12	0.07	0.11	0.12	0.11	0.08	0.11	0.04	0.02	0.06	0.00	0.03	0.02	0.03	0.02	0.09
BOVESPA					1.00	0.29	0.18	0.12	0.17	0.17	0.17	0.15	0.11	0.10	0.06	0.12	0.01	0.11	0.07	0.11	0.08	0.15
IPC						1.00	0.19	0.12	0.23	0.19	0.20	0.15	0.11	0.14	0.09	0.16	0.02	0.14	0.12	0.15	0.10	0.17
AEX							1.00	0.38	0.58	0.66	0.70	0.55	0.46	0.19	0.14	0.22	0.03	0.14	0.10	0.15	0.14	0.30
ATX								1.00	0.35	0.36	0.39	0.28	0.35	0.18	0.09	0.18	0.05	0.14	0.07	0.07	0.19	0.20
FTSE									1.00	0.52	0.59	0.50	0.40	0.20	0.13	0.22	0.02	0.16	0.11	0.14	0.17	0.28
DAX										1.00	0.70	0.50	0.51	0.16	0.12	0.19	0.01	0.11	0.10	0.14	0.10	0.28
CAC											1.00	0.54	0.55	0.16	0.12	0.19	0.01	0.13	0.09	0.13	0.13	0.30
SMI												1.00	0.37	0.16	0.16	0.20	0.02	0.12	0.10	0.14	0.15	0.25
MIB													1.00	0.14	0.09	0.14	0.01	0.07	0.05	0.06	0.11	0.22
HGSG														1.00	0.22	0.38	0.12	0.24	0.20	0.29	0.26	0.18
NIKKEI															1.00	0.23	0.01	0.12	0.14	0.24	0.25	0.13
STRTIM																1.00	0.06	0.25	0.26	0.29	0.26	0.20
SSEC																	1.00	0.03	0.04	0.02	0.04	0.03
BSE																		1.00	0.16	0.19	0.17	0.14
KLSE																			1.00	0.21	0.12	0.11
KOSPI																				1.00	0.18	0.15
ALLORD																					1.00	0.14

Table 8. Non-parametric tail dependence: τ_{np}

	SP	NASDAQ	TSX	MERVAL	BOVESPA	IPC	AEX	ATX	FTSE	DAX	CAC	SMI	MIB	HGSG	NIKKEI	STRTIM	SSEC	BSE	KLSE	KOSPI	ALLORD	Average
SP	1.00	0.81	0.47	0.18	0.34	0.50	0.38	0.25	0.35	0.44	0.38	0.31	0.34	0.19	0.15	0.22	0.07	0.17	0.09	0.15	0.18	0.30
NASDAQ		1.00	0.46	0.15	0.32	0.41	0.31	0.17	0.29	0.37	0.31	0.26	0.28	0.16	0.14	0.21	0.06	0.16	0.09	0.17	0.15	0.26
TSX			1.00	0.20	0.35	0.39	0.31	0.27	0.33	0.30	0.30	0.28	0.28	0.24	0.20	0.26	0.09	0.19	0.14	0.19	0.22	0.28
MERVAL				1.00	0.20	0.19	0.19	0.19	0.17	0.17	0.17	0.16	0.16	0.15	0.14	0.16	0.07	0.12	0.11	0.12	0.11	0.16
BOVESPA					1.00	0.38	0.27	0.23	0.24	0.25	0.25	0.22	0.22	0.20	0.16	0.22	0.09	0.18	0.13	0.15	0.17	0.23
IPC						1.00	0.29	0.26	0.30	0.29	0.27	0.22	0.24	0.21	0.16	0.22	0.11	0.19	0.15	0.19	0.15	0.26
AEX							1.00	0.35	0.60	0.52	0.73	0.55	0.45	0.23	0.19	0.28	0.09	0.17	0.14	0.15	0.19	0.32
ATX								1.00	0.37	0.36	0.40	0.31	0.36	0.25	0.18	0.30	0.10	0.21	0.17	0.16	0.24	0.26
FTSE									1.00	0.51	0.68	0.58	0.47	0.23	0.18	0.26	0.06	0.18	0.13	0.18	0.24	0.32
DAX										1.00	0.64	0.45	0.51	0.20	0.16	0.24	0.07	0.13	0.13	0.18	0.17	0.30
CAC											1.00	0.61	0.54	0.23	0.18	0.27	0.07	0.15	0.13	0.17	0.20	0.33
SMI												1.00	0.44	0.19	0.18	0.23	0.05	0.13	0.12	0.14	0.21	0.28
MIB													1.00	0.19	0.17	0.21	0.07	0.12	0.10	0.12	0.18	0.27
HGSG														1.00	0.30	0.52	0.17	0.32	0.23	0.35	0.32	0.24
NIKKEI															1.00	0.30	0.08	0.20	0.15	0.27	0.29	0.19
STRTIM																1.00	0.11	0.34	0.26	0.35	0.34	0.27
SSEC																	1.00	0.09	0.12	0.06	0.09	0.08
BSE																		1.00	0.19	0.21	0.24	0.19
KLSE																			1.00	0.16	0.17	0.15
KOSPI																				1.00	0.24	0.19
ALLORD																					1.00	0.21
